# Toward Zero‐Excess Alkali Metal Batteries: Bridging Experimental and Computational Insights

**DOI:** 10.1002/adma.202502052

**Published:** 2025-05-16

**Authors:** Pan He, Neubi Francisco Xavier, Matthias Johannes Golomb, Yupei Han, Yuheng Du, Qiong Cai, Yang Xu

**Affiliations:** ^1^ Department of Chemistry University College London London WC1H 0AJ UK; ^2^ School of Chemistry and Chemical Engineering Faculty of Engineering and Physical Sciences University of Surrey Guildford GU2 7XH UK

**Keywords:** computational approach, interface, metal plating/stripping, solid‐electrolyte interphase, zero‐excess

## Abstract

This review introduces alkali metal (Li, Na, and K) anode‐less and anode‐free batteries and conveys a synopsis of the current challenges regarding anode‐electrolyte interfaces. The review focuses on a critical analysis of the fundamental understanding of the (eletro)chemical and (electro)physical processes occurring at the anode, including metal nucleation and dendrite growth, the properties of liquid and solid electrolytes, and their roles in the metal stripping/deposition process and the formation of solid‐electrolyte interphase, and the properties of separators and their role in inhibiting dendrite growth. Solutions to tackle the challenges for anode‐less and anode‐free batteries are discussed extensively in the aspects of the modifications of the anode substrate, novel electrolyte solutions and SEI structures, interface design, and novel separators/solid‐state electrolytes to enable stable battery performances. To highlight the importance of bridging experimental and computational insights, experimental progress derived from a range of advanced characterization techniques is analyzed in combination with the advancement in multi‐scale theory and computational modeling. Finally, outlooks are provided from both experimental and computational points of view for the exciting field of zero‐excess alkali metal batteries.

## Graphical abstract

This review explores zero‐excess alkali metal batteries, highlighting anode–electrolyte interfaces, metal nucleation, dendrite growth, and SEI formation while comparing Li, Na, and K metals. It critically examines electrolyte and separator roles, emphasizing substrate design, electrolyte modifications, interfacial engineering, and solid‐state electrolytes. Experimental and computational advancements are integrated to guide future developments.

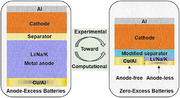

## 1 Introduction

In pursuit of next‐generation energy storage systems with high energy density, zero‐excess metal batteries (ZEMBs) have gained significant attention.^[^ ^1^ ^]^ These systems maximize the energy efficiency of metal‐based anodes while minimizing the use of inactive materials. ZEMBs are categorized into two main configurations. The first is the metal‐anode configuration, where a metal anode in an appropriate amount of metal pairs with a high‐capacity cathode, leveraging the energy density improvements from both components.^[^ ^2^ ^]^ For instance, conventional Li‐ion batteries (LIBs) utilizing a graphite anode (theoretical capacity: 372 mAh g⁻¹) and a LiFePO_4_ cathode (theoretical capacity: 170 mAh g⁻¹) can achieve an energy density of ≈250 Wh kg⁻¹. By replacing the cathode and anode with S (theoretical capacity: 1675 mAh g⁻¹) and Li (theoretical capacity: 3860 mAh g⁻¹), respectively, the theoretical energy density can reach ≈2500 Wh kg⁻¹—nearly ten times of that of conventional LIBs.^[^ ^3^ ^]^ The second is the anode‐free configuration, where a current collector on the anode side pairs with a charge carrier‐rich cathode, eliminating the weight of the anode material to enhance the energy density of the cell. For example, pairing a nickel‐cobalt‐aluminum (NCA) or nickel‐manganese‐cobalt (NMC) cathode with a Cu current collector can surpass the energy density ceiling of commercial LIBs (390 Wh kg⁻¹).^[^ ^4^ ^]^ Similar improvements can be achieved in ZEMB systems using other alkali metals, such as Na and K.^[^ ^5^ ^]^ The innovative designs of ZEMBs highlight their transformative potential in achieving unprecedented energy densities for future energy storage technologies.

However, the low potential of alkali metals (Li: 3.04 V, Na: 2.71 V, K: 2.93 V vs standard hydrogen electrode (SHE)) is a double‐edged sword.^[^ ^6^ ^]^ On the one hand, it increases the voltage and energy density of the full cell, but on the other hand, it enhances the reactivity of the metal anode, leading to a series of undesired reactions. For example, the intrinsically high chemical and electrochemical reactivity of alkali metals make them thermodynamically unstable, causing spontaneous reactions with the electrolyte upon contact.^[^ ^7^ ^]^ This results in electrolyte decomposition and the formation of solid‐electrolyte interphase (SEI) layers.^[^ ^8^ ^]^ Compared with the SEI formed on the graphite anode of LIBs and potassium‐ion batteries (PIBs)^[^ ^9^ ^]^ and the hard carbon anode of sodium‐ion batteries (SIBs),^[^ ^10^ ^]^ SEI layers on alkali metal surfaces are significantly more complex with much different composition and spatial distribution. Increasing evidence indicates that the SEI on alkali metal surfaces is inhomogeneous, unstable, and dynamically evolving.^[^ ^8^ ^]^ These characteristics lead to uneven ion transport and non‐uniform metal plating/stripping processes. Furthermore, the repetitive expansion and contraction of the metal anode during cycling make the SEI difficult to maintain, often resulting in fractures. The newly exposed metal surfaces react with the electrolyte, leading to further electrolyte consumption, reduced coulombic efficiency (CE), and the formation of non‐planar metal electrodeposition, all of which produce dendritic structures and/or electronically isolated regions (dead metals). The growth of dendrites can cause interelectrode short circuits, while the continuous consumption of electrolytes and accumulation of dead metals further decrease CE, ultimately leading to poor cycle life.

To address the challenges and advance the practical applications of ZEMBs, significant research efforts have been dedicated to understanding the formation and functionalization mechanisms of SEI, as well as exploring engineering strategies to stabilize interfacial reactions.^[^ ^11^ ^]^ Benefiting from the continuous development of advanced characterization methods, researchers have gained in‐depth insights into SEI components and structures at the nanometer scale and can now dynamically visualize the evolution of alkali metal plating/stripping at the nanometer‐to‐micrometer scale.^[^ ^12^ ^]^ Emerging imaging techniques such as cryogenic focused ion beam (cryo‐FIB) and cryogenic scanning transmission electron microscopy (cryo‐STEM) offer a unique opportunity to uncover the local structural and morphological details of SEI.^[^ ^13^ ^]^ However, experimental analytical techniques still have limitations in resolution, both spatially and temporally, especially when studying SEI formation, evolution, and alkali metal nucleation processes.^[^ ^14^ ^]^ Furthermore, experimental methods often produce “scattered” results that are specific to conditions. For instance, studies on Li metal batteries (LMBs) typically focus on a specific electrolyte or current density, providing insights that might not be able to represent a broad range of electrolytes/current density and other alkali metal batteries, e.g., Na and K metal batteries (SMBs and PMBs).

In this regard, theoretical calculations and simulation techniques provide a robust framework for understanding interfacial reactions, particularly during the early stage of alkali metal electrodeposition. Methods such as density functional theory (DFT), molecular dynamics (MD), and Monte Carlo (MC)/MD approaches are widely used to explore the atomistic‐level formation and the evolution of SEI.^[^ ^15^ ^]^ However, these techniques also face challenges, including the difficulty of defining appropriate boundary conditions due to the complexity of interfacial reactions and computational limitations in spatial and temporal scales. We can only achieve a more comprehensive understanding of interfacial reactions by integrating experimental and theoretical approaches. This synergy will enable the development of reasonable and effective solutions to mitigate side reactions at the interface, ultimately promoting the practical application of ZEMBs.

In this review, we begin by exploring the mechanistic understanding of SEI formation and evolution in alkali metals, emphasizing the commonalities and differences among Li, Na, and K metals. We then delve into ion diffusion across interfacial layers, focusing on key processes such as desolvation, ion transport within SEI, and metal plating on substrates. Particular attention is given to the critical roles of SEI and substrate in influencing metal nucleation. Subsequently, we summarize and compare dendrite formation—one of the most common and undesirable issues in alkali metal systems. Finally, we propose strategies to address current challenges, including anode structuring, electrolyte modification, artificial buffer coating layers, and new designs of SEI.

There exist excellent reviews on Li metal anodes and a few recent ones covering Na and K metal anodes separately,^[^ ^16^ ^]^ but comparative discussions among alkali metal systems remain scarce. This review fills this gap by systematically analyzing and at the same time, comparing interfacial reactions among Li, Na, and K systems. By integrating experimental observations with computational approaches, the review provides a comprehensive and mechanistic understanding of interfacial reactions and serves as a valuable reference for the development and optimization of alkali metal batteries and ZEMBs.

## 2 The Anode Reactions

**Figure 1:**
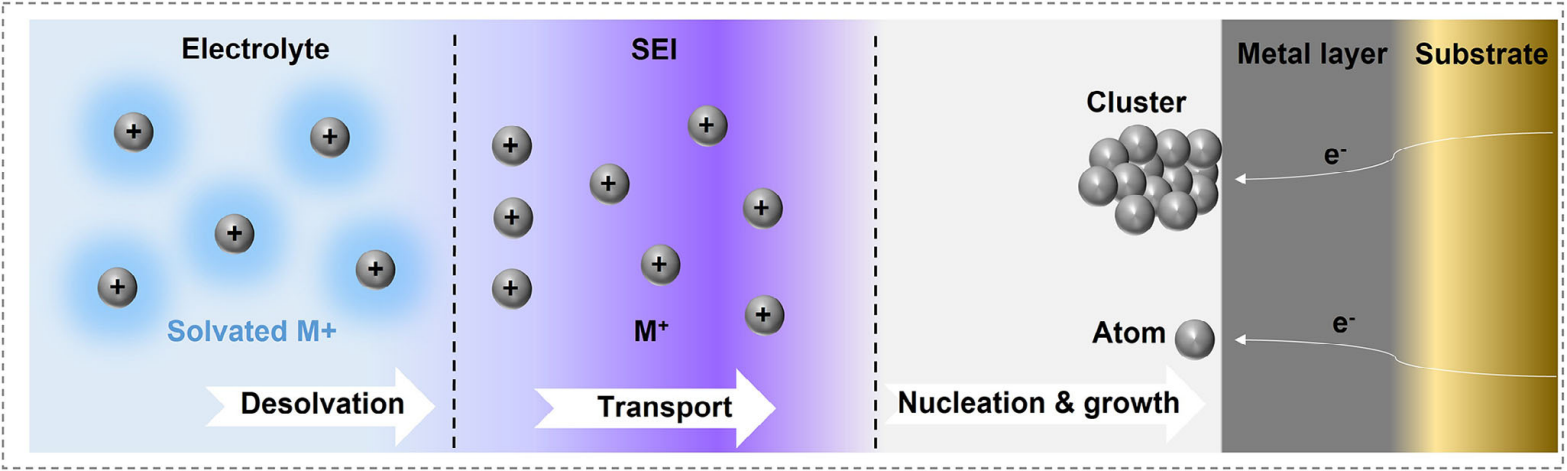
Schematic illustration of the anode reactions in alkali metal batteries. The deposition of alkali ions on the substrate contains three steps: desolvation of alkali ions (the left part), ion transport through SEI (the middle part), and nucleation and growth of alkali metals (the right part). The metal layer would be omitted for the anode‐free configuration.

The deposition of alkali ions in anode involves a series of interconnected processes that collectively determine the performance and stability of anodes (**Figure** **1**). Initially, ions approaching the anode surface must undergo desolvation, shedding their solvating molecules to interact with the solid surface, a step governed by an energy barrier that influences deposition kinetics. Once desolvated, the ions diffuse through the SEI, a passivating layer that regulates ion transport and prevents side reactions. The SEI's ionic conductivity and structural integrity are critical to ensuring uniform ion diffusion and deposition. Upon traversing the SEI, the ions undergo nucleation, clustering together to form stable nuclei on the current collector or pre‐existing sites, a process influenced by surface energy, substrate properties, and local ion concentration. Finally, these nuclei grow during the growth phase, where factors such as current density, ion flux, and electrolyte composition determine whether the growth is smooth and uniform or dendritic and uneven. Efficient and balanced progression through these steps is essential to achieving uniform deposition, maintaining electrode integrity, and preventing issues like dendrite formation that compromise battery performance and safety. The discussion in Section 2 will progress along the processes of the anode reactions.

### 2.1 SEI Formation and Evolution

Due to their strong reducing properties, the alkali metals Li, Na, and K readily react with various chemical species.^[^ ^17^ ^]^ Their application to batteries is no exception, and consequently, the appearance of interesting chemical reactions at the interface of alkali metal anodes and electrolytes was identified almost 50 years ago.^[^ ^7^ ^]^ With time, the so‐called “solid electrolyte interphase” has been identified as one of the most important parts of any battery cell, due to its importance for ion and electron transport across the battery – one could argue that the famous quote of Herbert Kroemer in his Nobel Lecture, “the interface is the device”, holds in this case as well.^[^ ^18^ ^]^ Despite this importance, the formation and evolution of the SEI remain one of the most challenging aspects of battery engineering due to its complexity. It for example strongly depends on electrolyte properties, the anode substrate, and the operation of the battery cell. Furthermore, the continuous stripping and plating processes upon charge and discharge mean that the SEI cannot be understood as a static component of the cell, but has to be seen as a dynamically evolving chemical interface.^[^ ^19^ ^]^

**Figure 2:**
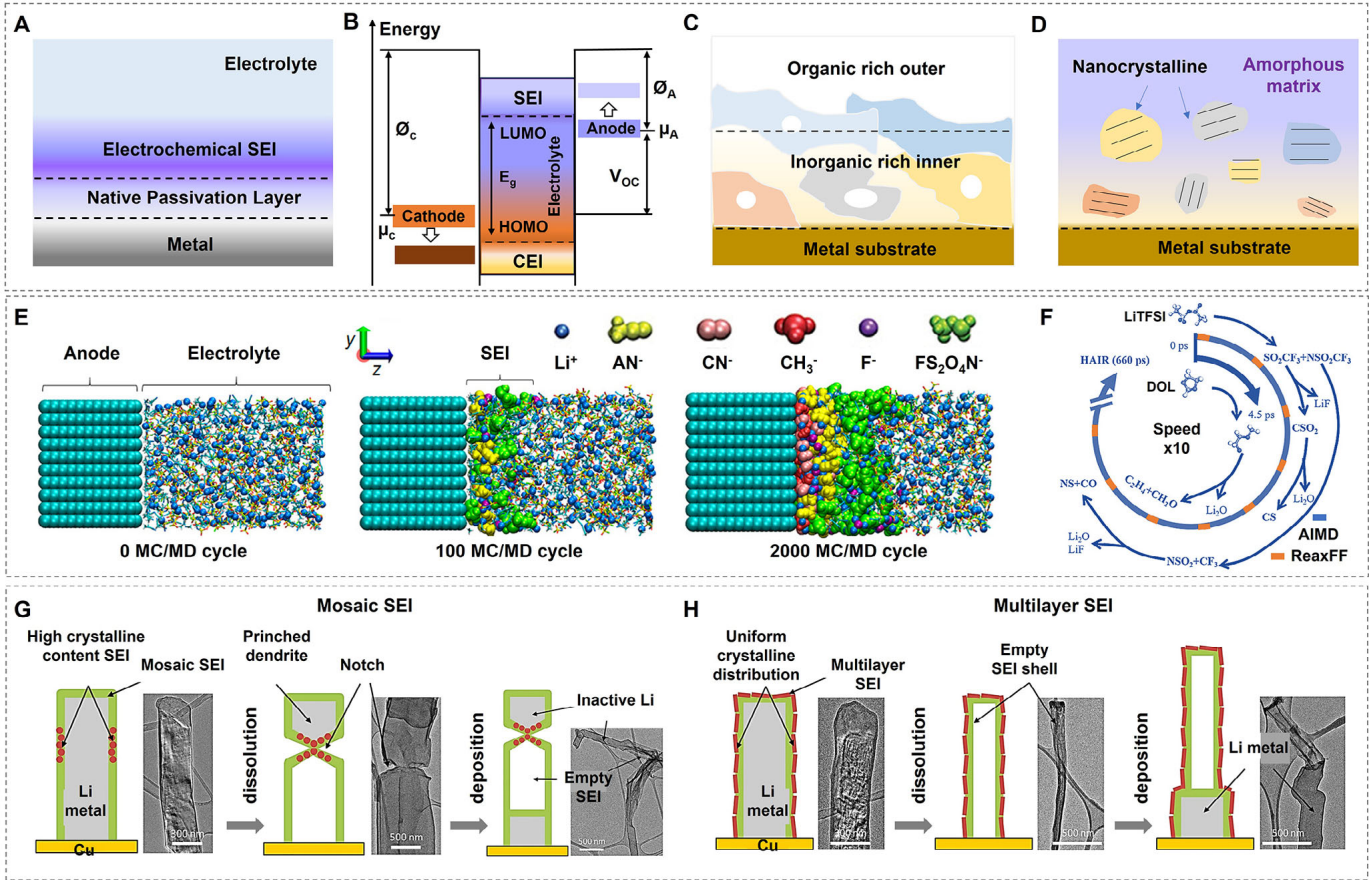
SEI formation and evolution. A) Schematic illustration of the surface structure on an alkali metal anode. B) Energy diagram showing the negative and positive potential requirements to ensure electrolyte stability. (Reproduced with permission.^[^ ^21^ ^]^ Copyright 2018, The Royal Society of Chemistry). C,D) Schematic illustrations of typical SEI structure distributions, showing variations in composition and morphology across the interface. E) Snapshots illustrating SEI formation processes in a highly concentrated LiFSA/AN electrolyte, showcasing temporal evolution. (Reproduced with permission.^[^ ^26^ ^]^ Copyright 2018, American Chemical Society). F) Reaction pathway for LiTFSI and DOL, obtained from HAIR simulations, detailing the intermediates and products. (Reproduced with permission.^[^ ^28^ ^]^ Copyright 2021, American Chemical Society). G,H) Comparison of Li stripping through different SEI structure: G) non‐uniform Li stripping facilitated by a mosaic SEI, and H) uniform Li stripping supported by a multilayer SEI, highlighting the impact of SEI architecture on electrochemical performance. (Reproduced with permission.^[^ ^22a^ ^]^ Copyright 2018 Elsevier Inc.).

When looking at the formation of the SEI, the first important aspect to consider is the starting structure of the two reactants responsible for its formation, namely the metal anode and the electrolyte. Due to the reactivity of alkali metals, the surface of alkali metal anodes is already covered by a native passivation layer even prior to contact with the electrolyte unless it has been explicitly removed (**Figure** **2**A).^[^ ^20^ ^]^ This layer is understood to originate from the reaction with air and is thus composed of Li_2_O, Li_2_CO_3_, and LiOH for Li batteries;^[^ ^17^ ^]^ while to the best of our knowledge, no works on the native passivation layer in Na/K metal anodes can be found, their higher reactivity leads to the expectation of the existence of a native passivation layer, formed of (Na/K)_2_O, (Na/K)_2_CO_3_, and (Na/K)OH by analogy. However, this native layer does not stop the response of the surface with electrolyte, as the layer lacks the necessary mechanical strength and density to suppress the occurring reactions. Recent evidence on Li metal anodes with solid‐state electrolytes also points to the native passivation layer having a continued detrimental effect on ion transfer upon cycling.^[^ ^20^ ^]^ In terms of starting structure, the initial state of the electrolyte also plays a crucial role, as its solvation structure influences the composition and distribution of species within the SEI. In general, species contained within the first ion solvation shell are thought to make up the majority of the SEI, which has sparked great research interest in high‐concentration electrolytes (HCE) and localized high‐concentration electrolytes (LHCE) as well as low‐concentration electrolytes. This is strongly connected to the desolvation process and will thus be explored further in Section 2.2.

Bringing the metal anode foil into contact with the electrolyte leads to the formation of another secondary layer on top of the native one, the electrochemical SEI, which suppresses further reactions of the two components after its formation. This interplay of battery components can be understood by considering the electronic structure of the anode, electrolyte, and cathode (Figure 2B). Fundamentally, the electrolyte is reduced under negative potentials by the anode and oxidized under positive potentials by the cathode.^[^ ^21^ ^]^ The electrochemical stability window of the electrolyte can then be defined as the energy difference between the potentials for reduction and oxidation: when the chemical potential of the anode µ_*A*_ is higher than the potential for the reduction of the electrolyte, the electrolyte is reduced; conversely, oxidation of the electrolyte will occur when the chemical potential of the cathode µ_*C*_ is lower than the potential for oxidation of the electrolyte. This means that for the SEI to suppress electrolyte reduction in terms of electronic structure, the SEI raises the reduction potential of the electrolyte, which in turn results in an increased electrochemical stability window of the electrolyte/SEI system. This steady state is however broken during charge and discharge when ions migrate from the electrolyte to the anode through the SEI and vice versa, and an applied potential changes the electrochemical potentials of the anode and cathode. From this energetical picture alone, we can deduce desirable properties of the SEI: it should be electronically insulating to suppress reduction, but ionically conductive to facilitate ion diffusion.

Throughout the years, many models have been proposed for the formation mechanism of the electrochemical SEI as well as the species composition and distribution within it. Recently, two of these models have been reported as directly observed using cryogenic electron microscopy: the multilayer as well as the mosaic model.^[^ ^22^ ^]^ In the former, the electrochemical SEI is proposed to consist of distinct layers of inorganic and organic decomposition products uniformly dispersed on top of an amorphous matrix (Figure 2C). The inner layer is inorganic‐rich, commonly consisting of MF, M_2_CO_3_, and M_2_O (M = Li, Na, K).^[^ ^23^ ^]^ The outer layer is organic‐rich, and its composition depends heavily on the electrolyte used for the cell; as an example, the most common electrolyte for LMBs, which is a mixture of 1,3‐dioxolane (DOL) and 1,2‐dimethoxyethane (DME) solvent, the O─CH_2_─O linkage‐containing decomposition products of DOL seem to dominate the organic SEI.^[^ ^24^ ^]^ The mosaic model on the other hand suggests that both inorganic and organic decomposition products are present as heterogeneously distributed crystallites within the amorphous matrix (Figure 2D). To investigate the conditions under which model structure is formed, Li et al. investigated LiPF_6_ in ethylene carbonate (EC) and diethyl carbonate (DEC) with and without fluoroethylene carbonate (FEC) additive, showing a mosaic SEI without FEC and a multilayer SEI with FEC.^[^ ^22a^ ^]^ Interestingly, their results suggest both initial SEI formations show similar dendrite morphology, but significant changes occur upon Li dissolution, where the electrolyte with FEC additive shows much thinner dendrites. This has been attributed to a more uniform SEI formation in the latter, preventing the creation of notches that lead to dead Li, ultimately explaining the improved cell performance of the FEC additive variant.

Due to the sensitivity of the SEI to characterization techniques and the very fast reaction times upon first contact, experimental validation of the initial formation process has proven challenging. This has motivated the use of theoretical techniques to describe the formation and evolution of SEI at an atomistic level. The most common approaches include DFT, MD, and MC/MD as well as ab initio MD (AIMD)/MD hybrid approaches. DFT is commonly used to calculate base properties of the chemical species participating in the formation of the SEI, e.g., the calculation of the highest occupied molecular orbital (HOMO) and lowest unoccupied molecular orbital (LUMO) of the solvent species to aid the calculation of the potentials for oxidation and reduction, or adsorption energies of solvent and salt moieties on top of the metal surface. MD on the other hand can be used to identify dynamic properties of the SEI but can reach computational limits due to the system size, complexity, and timescales needed for the description of the anode‐SEI‐electrolyte interface. Hybrid approaches such as the “Red Moon” method utilize MC calculations to describe long‐timescale processes and MD calculations to predict motions on shorter timescales.

One of the earliest studies on the formation of the SEI was carried out by Kim et al.^[^ ^25^ ^]^ They investigated the SEI formation for Li metal using EC as well as dimethyl carbonate (DMC) electrolyte using MD simulations with reactive force field (ReaxFF) potentials, showing Li_2_CO_3_ and Li_2_O formation on the anode surface for EC and LiOCH_3_ for DMC. However, no salts were present in the calculation, limiting the predictive power for real systems. Takenaka et al. employed the “Red Moon” method to study the SEI formation on carbon anodes (Figure 2E), considering both lithium bis(fluorosulfonyl)amide (LiFSA) salt and acetonitrile (AN) electrolyte at different concentrations.^[^ ^26^ ^]^ In a later study, they extended their approach to additionally take the electrode potential into account, leading to a high degree of representation of important cell operation properties.^[^ ^27^ ^]^ The HAIR (Hybrid ab initio molecular dynamics combined with reactive force fields) method proposed by Liu et al., in which classical force field MD and AIMD were alternated, showed great promise in maintaining AIMD accuracy while extending the simulation period to the nanosecond regime (Figure 2F).^[^ ^28^ ^]^ Yu et al. used this to investigate the formation of the SEI in lithium bis(fluorosulfonyl)imide (LiFSI)/fluorinated 1,4‐dimethoxybutane (FDMB) electrolytes and were able to explain its good performance in terms of the LiF‐rich inorganic inner layer thought to be beneficial for cell operation.^[^ ^29^ ^]^ Liu et al. applied the same method to Na batteries, studying possible solvent decomposition pathways of solvents and SEI formation products.^[^ ^30^ ^]^ Pure AIMD studies applied to metal anode/electrolyte interfaces are rare due to their computationally expensive nature; noteworthy examples are, e.g., the study of Stottmeister and Groß, in which the authors studied the decomposition of common electrolyte molecules such as EC and PC on Li, Na and K surfaces.^[^ ^31^ ^]^ They found the initial decomposition pathway to CO_3_ to be favorable for all systems, while the decomposition to CO was favorable only for K; comparing the decomposition energies between both pathways however showed that the carbonate product is the most favored in all cases.

While it might be more accessible for simulation to investigate the fast timescales and small spatial resolution of the initial SEI formation, the opposite is true for the evolution of the SEI upon cycling. Comparatively long timescales and effects on battery cell level limit the use of techniques such as DFT or AIMD, although they can still give insight into the energetics of possible reactions during the evolution, like in the work of Liu et al. on ether solvents.^[^ ^24^ ^]^ As such, experimental techniques such as nuclear magnetic resonance (NMR) or time‐of‐flight secondary ion mass spectrometry (TOF‐SIMS) offer avenues to investigate the state of the SEI after multiple cycles.^[^ ^32^ ^]^ For example, the latter was employed by Ma et al. together with XPS species analysis to study the SEI evolution of LiPF_6_ EC/DMC electrolyte cells as a function of different formation currents, indicating that intermediate current densities lead to better performance results than both low and high ones. This was attributed to an optimal balance between SEI and Li conduction channels through which cycling can occur, emphasizing the importance of the initial structure of the SEI for the cycles thereafter. The state of the initial SEI formation was also identified as critical in the cryo‐EM studies of Li et al.^[^ ^22a^ ^]^ A uniform SEI led to a uniform dissolution and re‐deposition on the dendritic anode surface, suppressing the creation of dead lithium during the evolution upon cycling (Figure 2G–H).^[^ ^22a^ ^]^ The results obtained for Li metal anodes can be valuable for investigating the SEI of Na and K metal anodes, given that studies have suggested their SEIs to be less stable upon cycling or even self‐dissolving after formation compared with the Li counterpart.^[^ ^33^ ^]^

### 2.2 Desolvation of Alkali Metal Ions

As mentioned before, the electrolyte itself is a crucial battery component determining both the SEI formation and evolution as well as the overall performance of the cell due to the relation of the electrolyte potentials for reduction and oxidation to the overall cell voltage. The former is closely connected to the process of desolvation at the electrolyte/SEI interface, in which the ion loses its solvation sheath to be first adsorbed on top of the SEI surface and consequently be absorbed into the SEI where it diffuses to the anode. This has sparked great research interest in electrolyte engineering for SEI optimization, utilizing electrolytes that incorporate beneficial components in their first solvation sheath. Generally, the alkali metals differ in their Stokes radii in the order of *r_S,Li_ > r_S,Na_ > r_S,K_*, which leads to the trend of higher ionic mobilities of Na and K ions in electrolytes and different desolvation energies.^[^ ^34^ ^]^

**Figure 3:**
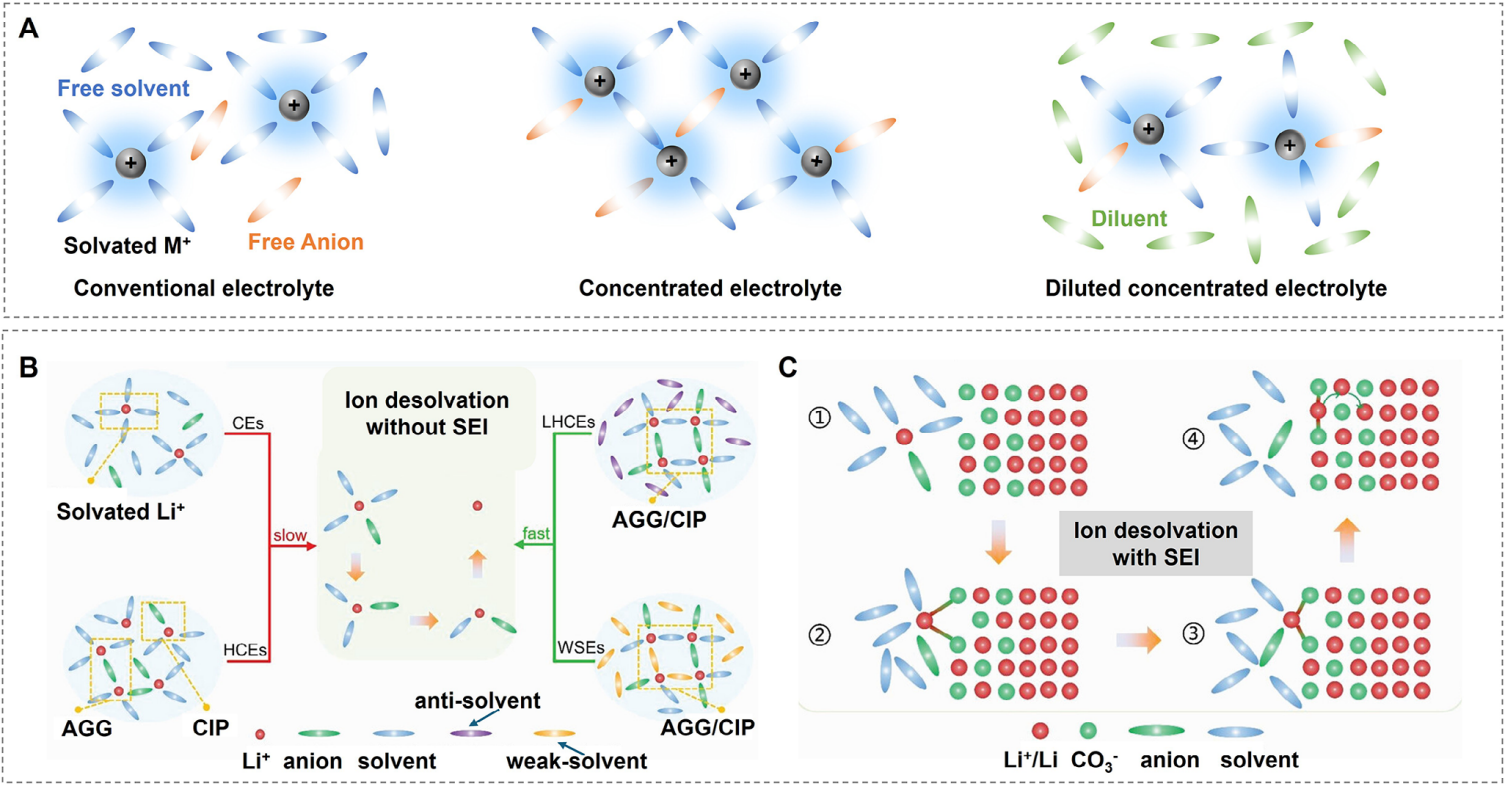
The desolvation process without/with SEI involvement. A) Schematics of the solution structures of electrolytes: conventional dilute electrolyte, concentrated electrolyte, and diluted concentrated electrolyte. The concentrated electrolyte retains its unique local coordination structure even after dilution with a low‐polarity solvent (diluent). (Reproduced with permission.^[^ ^35^ ^]^ Copyright 2019, Springer Nature Limited). B,C) Comparison of conventional and newly proposed models of ion desolvation: B) conventional ion desolvation model, considering only the electrolyte and neglecting the role of the SEI in the desolvation process, C) newly proposed ion desolvation model at the electrolyte/SEI interface, considering the influence of the anions and the SEI whose active sites facilitate ion desolvation by “catching” or associating ions. (Reproduced with permission.^[^ ^36^ ^]^ Copyright 2023, Wiley‐VCH GmbH).

Conventional electrolytes usually exhibit a first solvation sheath where ions coordinate with solvent molecules only (**Figure** **3**A).^[^ ^35^ ^]^ HCEs however gathered attraction due to the exchange of solvent molecules in the first solvation sheath with salt anions. This commonly results in an SEI that is dominated by inorganic domains created from salt anion decomposition products. Localized high‐concentration electrolytes seek to overcome two major drawbacks of high‐concentration electrolytes, namely high viscosity and the increased cost of HCE fabrication. A weakly solvating solvent, also called diluent, addresses these points due to it not coordinating with the ions, keeping the same local ion structure as its HCE counterpart (Figure 3B).^[^ ^36^ ^]^ This results in a SEI that is still dominated by inorganic species, but shows much faster desolvation kinetics.

In 2018, Chen et al. showed the beneficial properties of LHCEs with the example of bis(2,2,2‐trifluoroethyl) ether (BTFE) in LiFSI/DMC electrolyte.^[^ ^29b^ ^]^ They reported improved SEI morphology as well as reduced SEI thickness, leading to high Coulomb efficiencies and cyclability. For Na metal batteries, Zheng et al. showed that the addition of BTFE to NaFSI/DME electrolyte improved performance greatly, leading to a remarkable capacity retention of 90.8% after 40 000 cycles.^[^ ^37^ ^]^ Similar improved battery cell properties with electrolyte engineering were also demonstrated in a study by Zhao et al.^[^ ^38^ ^]^ They employed the weak‐coordinated diluent bis(2,2,2‐trifluoroethoxy) methane (BTFM) in DME instead of a non‐coordinating diluent, which had been commonly used for LHCEs, which led to a Li_2_O‐rich SEI. Using LiFSI as the salt, their experiments found exceptional cell performance in terms of electrochemical stability and Coulomb efficiencies of up to 99.72%. In a recent study, Cheng et al. even used a trisalt electrolyte comprised of FEC and DMC solvent with high concentrations of LiTFSI, LiPF_6_, and LiNO_3_ additives.^[^ ^39^ ^]^ This led to a SEI with comparatively little organic contribution, resulting in improved smooth Li deposition morphology and enhanced cyclability for the cell. Similarly, the addition of perfluorobenzene (PFB) to NaPF_6_/EC/PC electrolytes resulted in a NaF‐rich SEI in the study of Zhu et al.^[^ ^40^ ^]^ This was explained by a change in solvation sheath, which without the additive shows only little coordination of PF_6_ to Na due to its higher solvation energy. Analogously, even the simple addition of water molecules, as demonstrated by Sun et al. for diethylene glycol dimethyl ether (DEGDME)/LiTFSI electrolyte, can change the ion solvation sheath to contain more salt anions, leading to a low‐cost approach of improved SEI formation.^[^ ^41^ ^]^

The successes of electrolyte engineering are obvious; due to the extremely small time and length scales as well as the sensitivity of the dynamic process to characterization techniques, simulations are crucial to the understanding of atomistic mechanisms behind improved cell performances. The desolvation process can be seen as a decomposition reaction with multiple energy barriers corresponding to breaking the coordination of a single component of the first solvation sheath. Interestingly, the binding energy of salt anions is usually much larger than that of the solvent – this should result in a higher energy barrier to dissolve the ion in (L)HCEs. This has been challenged by calculations of binding energies in solution instead of in vacuum, where the calculations of Chen et al. indicated lower binding energies for PF_6_ in both DME and EC solvents.^[^ ^42^ ^]^ It has also been suggested that the SEI itself acts as a catalyst for the salt anion desolvation, resulting in a distinct order of steps for the process: Initially, the solvent coordination is broken, and the ion coordinates to the SEI instead. This then catalyzes the dissolution of the salt anion coordination (Figure 3C). Wang et al. studied these effects using the example of Li_2_CO_3_ as the SEI component, which interacted favorably as a catalyst for desolvation in both LiDOL_2_FSI and LiEC solvation structures.^[^ ^36^ ^]^

It has also been noted that ion transport through the SEI is not independent of the direction of diffusion, meaning that a distinction must be made between desolvation and solvation of the ion in order to compare energies obtained in the experiment to computational results. Tanibata et al. showed that this is an experimentally often overlooked feature due to the averaging of charging/discharging processes with alternating current impedance spectroscopy. They used a Laplace transform impedance method to separate the resistances of the two processes and identified the solvation resistance to be higher than the desolvation resistance for three commonly used salts in a propylene carbonate electrolyte.^[^ ^43^ ^]^

### 2.3 Ion Transport in the SEI

The ion transport behavior of SEI is a crucial property for every battery since it governs the deposition of ions on the anode during continued cycling. It has been suggested that a uniform, evenly spread transport of ions leads to less dendrite formation and good cycling behavior, whereas heterogeneous transport results in uneven plating and ultimately the loss of active material.

**Figure 4:**
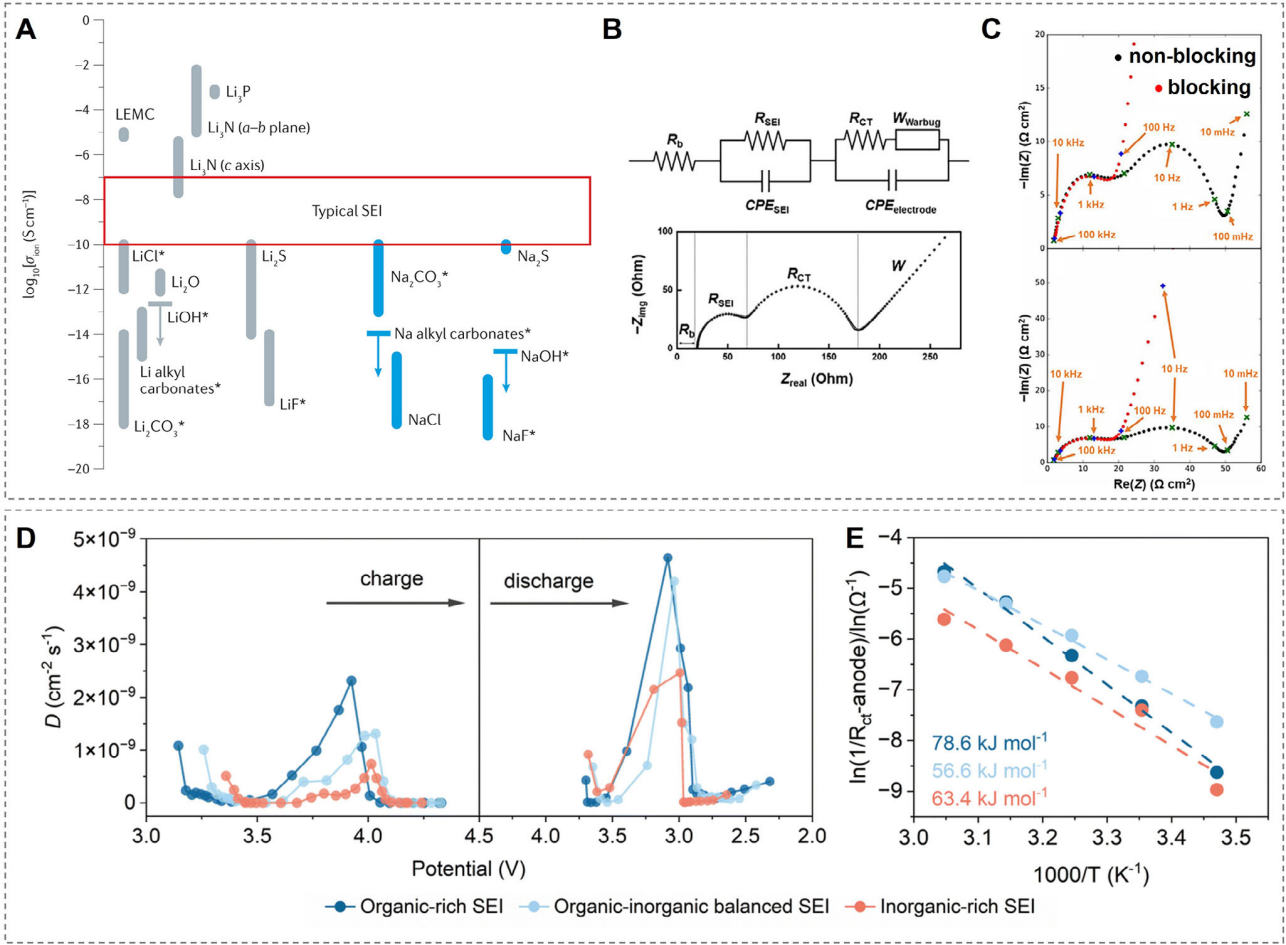
The ion conductivity of typical components of the SEI. A) Ionic conductivity of SEI components at 25 °C, measured from single‐phase bulk samples. (Reproduced with permission.^[^ ^44^ ^]^ Copyright 2021, Springer Nature Limited). B) Equivalent circuit model of a Li‐ion battery half‐cell. Key elements include: *R_b_* (bulk resistance of the cell, including the resistance from the electrolyte, separator, and electrodes), *R_SEI_* and *CPE_SEI_* (resistance and capacitance of the interfacial layer), *R_ct_* and *CPE_electrode_* (charge‐transfer resistance and double‐layer capacitance), and *W_Warburg_* (diffusional effects of Li^+^ on the host material). (Reproduced under the terms of the Creative Commons Attribution Non‐Commercial License.^[^ ^45^ ^]^ Copyright 2020, Open access). C) Typical area‐specific Nyquist plots of blocking and non‐blocking full coin cells measured at 20 °C, with labeled frequency decades. (Reproduced with permission.^[^ ^46a^ ^]^ Copyright 2019, The Author(s), Published by ECS). D) K^+^ diffusion coefficients at various voltages for full cells derived from GITT curves. E) Activation energies of the *R_ct_*‐anode at ≈4.5 V for full cells. (Reproduced with permission.^[^ ^47^ ^]^ Copyright 2025, The Royal Society of Chemistry).

Loosely aligned with the two models of the SEI, the mosaic, and the multilayer model, we can assign the following transport regimes to the components of the interphase: I) Transport in the organic material, which can be divided into ordered and amorphous domains as well as their grain boundaries, II) transport in the inorganic material, which can be divided into ordered crystalline domains of extended layers or crystallites and their grain boundaries, and III) transport in the grain boundaries of organic and inorganic domains. While these regions generally show differences, in terms of fundamental mechanisms of ionic transport they can be broadly categorized into either ordered or disordered systems. The former has been studied for many materials and applications and multiple underlying principles have been identified, whereas much less effort has been afforded to study disordered SEI systems which might be a possible explanation for the difference in calculated ion conductivities and those measured in typical SEIs (**Figure** **4**A).^[^ ^44^ ^]^ Studies on Li metal anodes and their SEI materials are much more common than those for Na metal systems, while very few K metal anode SEI transport studies have been reported. Due to similarities in the SEI structure, however, results from studies on other anode systems can also be indicative of metal anodes.

In situ experimental characterization of transport in the SEI is once again challenging due to the reasons mentioned before, which is why most experimental reports focus on electrochemical impedance spectroscopy (EIS) to measure the ionic conductivity across the SEI by means of an equivalent circuit model (Figure 4B).^[^ ^45^ ^]^ The challenge for interpretation lies then in the separation of the impedance of the different components of the model. For example, Keefe et al. performed temperature‐dependent EIS to separate the influence of charge transfer and contact impedance in Li battery SEIs (Figure 4C), whereas Li et al. attempted to distinguish between contributions of the desolvation and the actual transport step by comparison to lithium titanate systems, which do not favor SEI formation.^[^ ^46^ ^]^ They concluded that no general remarks about the dominating step can be made, but that the rate‐determining one is highly dependent on electrolyte and anode chemistry. In a rare study on K ion diffusion through the SEI, Mo et al. focused on the contribution of the organic layer to transport kinetics (Figure 4D,E).^[^ ^47^ ^]^ They employed in situ EIS and an analysis of the distribution of relaxation time (DRT) to systematically probe the effect of SEI composition on diffusion coefficients and activation energies, which indicated optimal performance at a balance of organic and inorganic materials, contrary to the common conception of improved performance of inorganic dominated SEIs.

**Figure 5:**
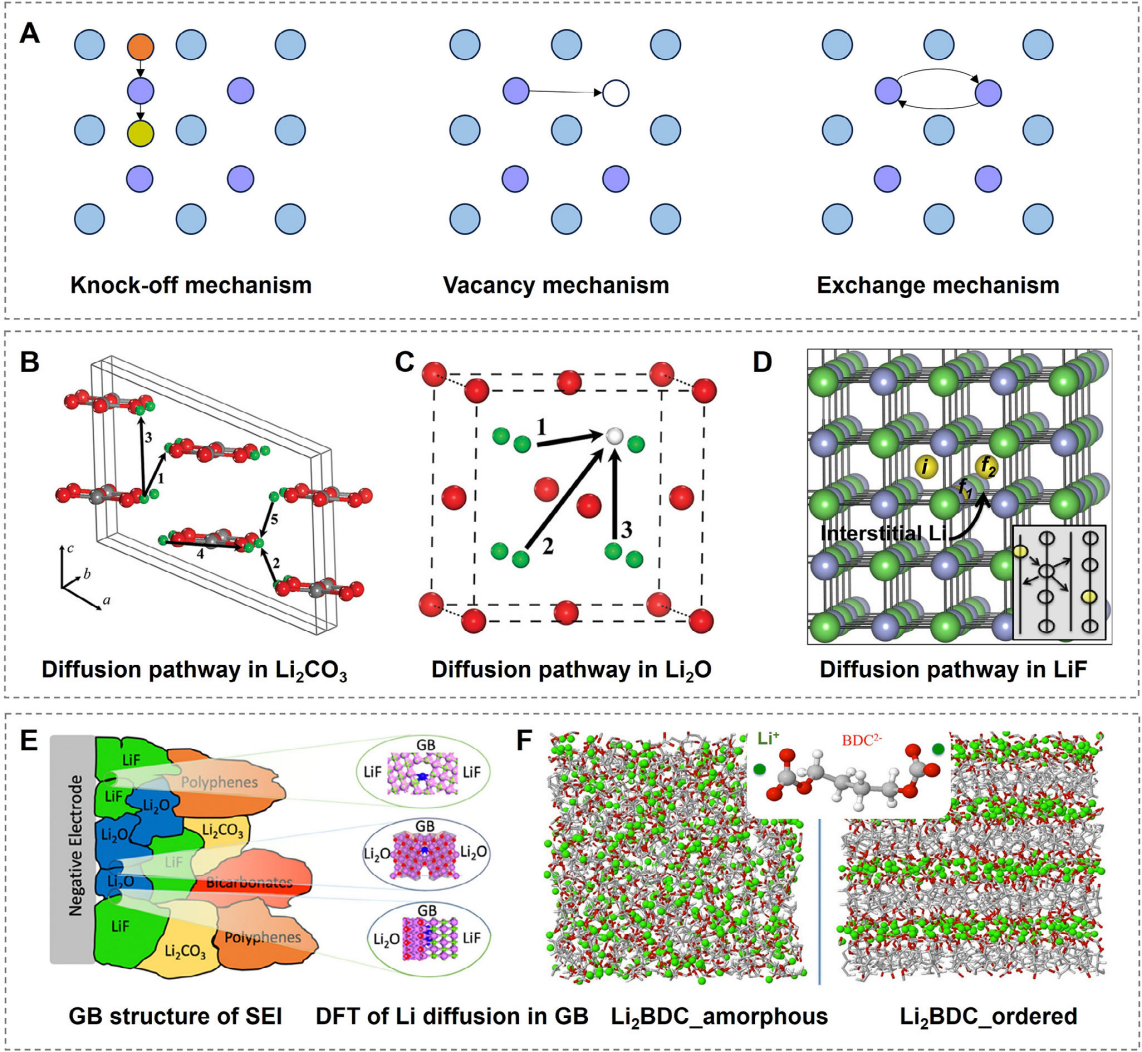
Ion transport mechanisms in typical SEI components. A) Schematic illustrations of the typical Li‐ion transport mechanisms in the inorganic components of SEI: (left) knock‐off mechanism, (mid) vacancy mechanism, (right) direct exchange mechanism. (Reproduced with permission.^[^ ^48^ ^]^ Copyright 2024, Elsevier Ltd.). B–D) Li^+^ transport mechanisms in typical inorganic SEI components. B) Li₂CO₃ via the vacancy mechanism operating under high voltage (gray, red, and green spheres represent C, O, and Li atoms, respectively); C) Li₂O via the vacancy mechanism operating at high temperatures (red and green spheres represent O and Li atoms, while white spheres indicate Li vacancy positions) (Reproduced with permission.^[^ ^51^ ^]^ Copyright 2011, American Chemical Society). D) LiF via the knock‐off mechanism (gray, green, and yellow spheres represent F, Li, and interstitial Li, respectively). (Reproduced with permission.^[^ ^53^ ^]^ Copyright 2011, American Chemical Society). E) Li^+^ transport in complex SEI compounds: transport mechanism in Li₂O/Li₂CO₃. (Reproduced with permission.^[^ ^54^ ^]^ Copyright 2020, American Chemical Society). F) MD simulation results show modulus and ion conductivity calculations of organic SEI compounds under disordered and ordered conditions and highlight the structural influence on transport properties. (Reproduced with permission.^[^ ^57^ ^]^ Copyright 2017, American Chemical Society).

Due to most experimental characterization only being available post‐cycling, and the difficulty of separating various fundamental transport contributions, simulation offers an avenue to study the microscopic processes responsible for the macroscopic effects seen in the experiment. In the simulation, ion transport in ordered systems is usually categorized into interstitial, knock‐off, vacancy and direct‐exchange mechanisms (**Figure** **5**A).^[^ ^48^ ^]^ In the interstitial mechanism, an absorbed ion at an interstitial site directly occupies a neighboring, free interstitial site. This type of transport is often associated with mobile ions smaller than the lattice atoms since the occupation of interstitials with larger species would induce significant lattice strain. The knock‐off mechanism describes an absorbed ion at an interstitial site displacing a lattice atom at a lattice site, which then moves on to become a “free” atom at an interstitial site itself. Vacancy transport occurs due to the intrinsic existence of crystal vacancies at finite temperatures, which a transporting ion can occupy. In direct exchange, two neighboring lattice site atoms switch positions. This collective movement is considered defect‐free since it does not involve interstitial or vacant sites. Identifying the dominant transport mechanism can provide valuable insights for experimentally improving the SEI. If the dominant transport type is predicted to be defect‐independent (such as direct exchange), defect engineering may be less critical. Conversely, if transport relies on defects, targeted defect engineering strategies can be applied to enhance ion conductivity and stability.^[^ ^49^ ^]^

Usually, these mechanisms are not found purely within one material but compete, thus their relative frequency depends on the activation barrier for each process (Figure 5B–D). Shi et al. determined the diffusion in Li_2_CO_3_ to be a knock‐off mechanism of Li^+^ by rigorous application of defect formation theory, considering the change in chemical potential for the cell upon cycling, and subsequent nudged elastic band (NEB) calculations.^[^ ^50^ ^]^ They attributed the favorable energetics of knock‐off vs interstitial transport to the increased coordination of Li^+^ to oxygen along the diffusion pathway in the former. They also found the ion conductivity in this material to be highly anisotropic, which emphasizes the orientation of Li_2_CO_3_ crystallites/layers within the SEI. NEB calculations were also employed in the study of Chen et al.,^[^ ^51^ ^]^ which indicated the migration barrier in LiF to be much higher than those in Li_2_O and Li_2_CO_3_, which were confirmed by MD calculations of Benitez et al. in their study of Li mobilities over wide temperature ranges.^[^ ^52^ ^]^ Yildirim et al. however reported that similar to Li_2_CO_3_, a knock‐off diffusion in LiF shows a considerably lower barrier of 0.27 eV in LiF, which is similar to the barrier heights of other SEI components.^[^ ^53^ ^]^ They also calculated diffusion barriers for transport in NaF, which proved to be considerably higher than LiF in agreement with experimentally reported ion conductivity values. Interestingly, the study of Li transport at crystalline grain boundaries of LiF and Li_2_O using DFT and NEB by Ramasubramanian et al.^[^ ^54^ ^]^ (Figure 5E) found activation barriers as low as 0.48 eV, suggesting that grain boundaries could be another preferred pathway for ion transport in the SEI. These findings have been confirmed by calculations of Ma et al., which reported high conductivity values obtained through NEB calculations at the grain boundaries of LiF/Li_2_O, Li_2_O/Li_2_CO_3,_ and LiF/Li_3_N.^[^ ^55^ ^]^ Experimental verification of these computational diffusion parameters is challenging due to the multiphase and dynamic nature of the SEI. However, a recent study by Guo & Gallant successfully synthesized single‐phase SEIs of Li₂O and LiF grown on Li foils.^[^ ^56^ ^]^ Their findings showed good agreement with computationally predicted values and further confirmed the impact of formation temperature on ion conductivity. This effect is attributed to changes in defect concentration, grain size, and the presence of grain boundaries.

Very few fundamental principles have been established for the diffusion of ions in disordered SEI layers. MD simulations on LEDC and LBDC indicate however that at low temperatures, ion and anion mobilities are decoupled from each other with the anion matrix being frozen, and the ion mobility is solely determined by the existence of percolating channels of high ion concentration through the material (Figure 5F).^[^ ^57^ ^]^ Jorn et al. investigated the diffusion of Li ions in disordered LEDC and crystalline Li_2_CO_3_ in EC/LiPF_6_ electrolytes with MD.^[^ ^58^ ^]^ By examining the coordination of Li ions to EC, they were able to monitor the desolvation at the SEI interface as well as the diffusion within the SEI, showing a clear two‐barrier process consisting of the energy cost associated with desolvation and then migration through the SEI. Interestingly, they also found that the insertion and extraction of ions behave differently: for insertion, their calculations suggested that the coordination of the ion to SEI surface carbonate groups occurs directly at the surface upon loss of EC coordination. During extraction, however, the ion retains coordinated carbonate groups deeper into the electrolyte, bending and rotating EDC until the exchange of coordination to EC occurs and EDC retreats to the SEI surface.

### 2.4 Metal Plating on Substrates

Similar electrochemical mechanistic principles govern alkali metal deposition on Li/Na/K metal batteries after migration through the SEI layer: reduction and clustering/nucleation. In particular, the morphology of the initial nucleation of Li/Na/K on the substrate has a decisive impact on determining their subsequent growth morphology during cycling. However, its mechanism remains inconclusive due to the short duration of the nucleation stage, the small size, and the varying shapes of the alkali metal nuclei, which depend on the adopted substrate.^[^ ^59^ ^]^ The current understanding of alkali metal nucleation follows the heterogeneous nucleation theory, in which deposited atoms form aggregates with a constant critical spherical radius, independent of the substrate. However, from a thermodynamic perspective, the formation of a new nucleus alters the surface energy, affecting the overpotential barrier and potentially varying significantly depending on the substrate. A high binding energy of the substrate towards the alkali metal corresponds to a low wetting angle, resulting in a small nucleation volume of the metal, benefitting the uniform distribution of metal nuclei and promoting dendrite‐free deposition.^[^ ^59^, ^60^ ^]^ Therefore, substrates with high binding energy towards the alkali metal are directly related to lower nucleation overpotential and are one of the most used descriptors for evaluating the effectiveness and uniformity of metal nucleation on a substrate. Cu and Al are the most used substrates for Li/Na/K metal deposition. Yet, the direct deposition of metal ions on their surfaces results in noticeable metal nucleation overpotential losses, which are associated with higher nucleation barriers and thereby affect the metal deposition morphology.^[^ ^61^ ^]^ This highlights the importance of investigating different substrates that promote a lower nucleation barrier for the initial alkali metal nucleation to achieve uniform Li/Na/K deposition.

**Figure 6:**
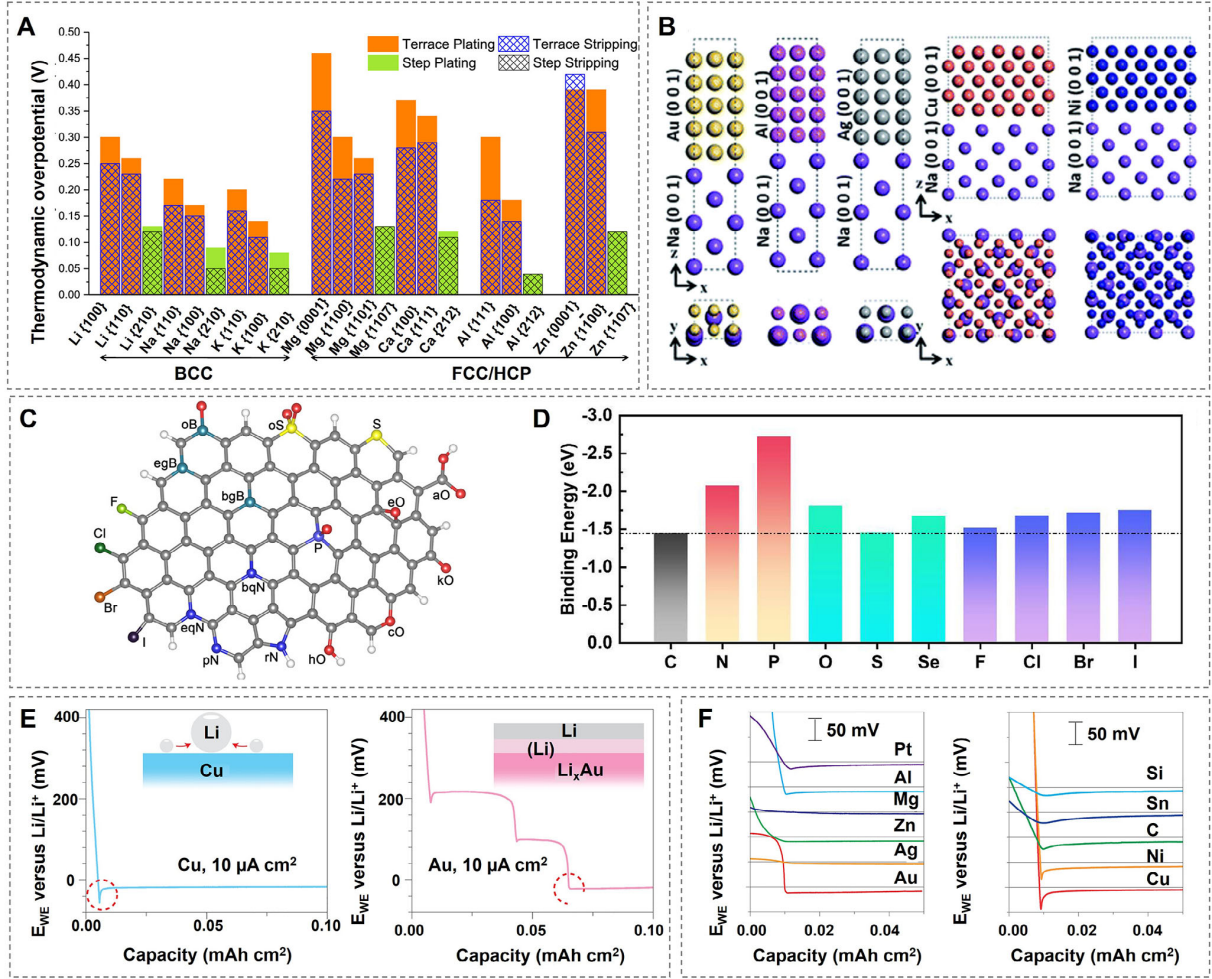
Metal plating on substrates. A) Thermodynamic overpotentials for electrodeposition and dissolution of seven metals, calculated as a function of surface facet and surface morphology (terraces vs steps). (Reproduced with permission.^[^ ^62^ ^]^ Copyright 2019, American Chemical Society). B) Atomic structures of fully relaxed supercells for various metal/alkali metal interfaces, including Au(001)/Na(001), Al(001)/Na(001), Ag(001)/Na(001), Cu(001)/Na(001), and Ni(001)/Na(001), shown in (left) top and (right) side views. (Reproduced with permission.^[^ ^61a^ ^]^ Copyright 2019, The Royal Society of Chemistry). C) Schematic of the of possible binding sites for Li atoms in heteroatom‐doped C single layer. (Reproduced with permission.^[^ ^64^ ^]^ Copyright 2019, The American Association for the Advancement of Science). D) Illustrations of the binding energy between K atoms and carbon materials doped with nonmetallic elements (e.g., N, O, S, P, F). (Reproduced with permission.^[^ ^60a^ ^]^ Copyright 2024, American Chemical Society). E) Voltage profiles of galvanostatic Li deposition on Cu and Au substrates at 10 µA cm⁻^2^, illustrating the influence of substrate material on deposition behavior. F) Voltage profiles of materials with varying solubility in Li during deposition at 10 µA cm⁻^2^, with curves horizontally shifted for Li nucleation onset and vertically shifted by 0.05 V for enhanced comparison. (Reproduced with permission.^[^ ^73^ ^]^ Copyright 2016, Springer Nature Limited).

Atomistic modeling based on DFT can reproduce binding energies with high accuracy and serves as a cost‐effective tool to screen different substrates for metal plating. Nagy et al. conducted a computational screening of overpotentials associated with the plating and stripping processes on various substrates,^[^ ^62^ ^]^ including Li, Na, K, Mg, Ca, Al, and Zn. The largest overpotentials were observed for the deposition of Ca and Mg, while the most efficient deposition occurred on the stepped surfaces of bcc metals (**Figure** **6**A), leading to higher nucleation rates. The limiting step for both deposition and stripping was identified as the first step on terrace surfaces and step edges, which largely determines the overpotential of these mechanisms. The metal/substrate interface strength was used as a descriptor for low overpotentials during Na nucleation in seawater batteries, as demonstrated by Jung et al.^[^ ^61a^ ^]^ Work of adhesion and binding energy values were estimated for Na/Au, Na/Ag, Na/Cu, Na/Al, and Na/Ni interfaces using DFT simulations (Figure 6B). The strongest interface adhesion was observed for Na/Au, while Na/Ni exhibited the highest binding energy. These findings were applied to promote patterned growth of Na metal islands on Al substrates, improving the cycling capability and electrochemical performance of the seawater battery.

Advanced experimental techniques, such as high‐resolution TEM, have been used to capture the initial nucleation steps of materials at the nanoscale, even enabling the identification of metastable structures.^[^ ^63^ ^]^ However, due to the highly dynamic nature and smaller radii of Li, Na, and K atoms, the mechanisms of formation and early growth of these metal clusters, which can vary depending on modifications and functionalization of the substrate, are difficult to characterize. In this context, DFT calculations are highly valued for providing mechanistic insights into substrate modifications aimed at increasing the binding between the substrate and alkali metals. Among these strategies, the introduction of Li/Na/K‐philic sites (i.e., sites with strong binding between the substrate and metal) is a key approach to promote the uniform deposition of alkali metals on substrates. The generation of new Li‐philic sites on heteroatom‐doped carbon substrates for Li deposition in lithium metal batteries was investigated by Chen et al. using a combination of first‐principles calculations and experimental measurements of overpotential.^[^ ^64^ ^]^ Based on their findings, general insights into the creation of Li‐philic sites were proposed. These included the formation of electronegative sites on doped atom‐carbon pairs and the establishment of strong local dipoles to enhance metal binding on doped atoms and facilitate efficient charge transfer. Following these principles, the co‐doping of O with B, S, or P resulted in stronger Li binding and reduced nucleation overpotential compared to single dopants (Figure 6C). In a subsequent work, Chen et al. investigated, using the same methodologies, the effect of doped carbon materials for Na and K metal batteries.^[^ ^65^ ^]^ The authors reported similar trends in binding energy estimations, suggesting that the principles established earlier are effective for designing Na‐philic and K‐philic sites.

Na‐philic sites were introduced on porous nitrogen‐doped carbon polyhedrons by embedding single Co atoms, as demonstrated in the work of Li et al.^[^ ^66^ ^]^ The increase in binding strength was confirmed by DFT calculations, and the strong affinity between Co and Na promoted spatial control of Na deposition, inhibiting dendrite growth. Tang and co‐workers conducted DFT and experimental investigations to design a DN‐MXene sheet that acted as potassium‐philic seeds, facilitating the organized nucleation of K atoms in K‐metal batteries (KMBs).^[^ ^67^ ^]^ The morphology of potassium‐deposited scaffolds was observed through SEM images, which revealed dendritic structures for direct K deposition on Cu and Al current collectors. However, the application of DN‐MXene/CNT scaffolds on the substrate surface acted as K‐philic sites, enabling the formation of metallic K within the porous structure, confining dendritic growth, and significantly improving cycle life. A doping strategy based on DFT workflow was employed to promote the generation of K‐philic sites in potassium metal batteries, as described in the work of Chen et al.^[^ ^60a^ ^]^ A systematic screening of various nonmetallic atoms doped in carbon skeletons revealed the highest binding strength with P‐doped carbon (Figure 6D). P‐doped carbon nanofibers were designed, and experimental observations indicated the formation of spherical K nuclei, facilitating the uniform deposition of potassium. Overall, the combination of DFT techniques and experimental studies has been effective in guiding substrate modifications to enhance metal‐philicity, reduce the nucleation energy barrier, and provide insights into the mechanisms of initial metal nucleation.

Alloying substrates can provide an additional migration pathway for alkali metals towards the substrate, potentially avoiding the standard surface plating observed in non‐alloying current collectors such as Cu.^[^ ^68^ ^]^ Electrochemical alloying reactions fall into two classifications: solid solution alloy reactions or reconstitution reactions that form intermetallic alloys. In the former, the reaction can be modeled as A + xM ⇋ AM_x_​, where no phase or structural change occurs in substrate M when the alkali metal (A) enters its structure.^[^ ^69^ ^]^ For a reconstitution reaction, the alkali metal A reacts with the element M to form a new compound MN_y_, in which N represents a new component displaced from the parent phase. The ability of metals that form intermetallic alloys with Li/Na/K to accommodate more than one alkali metal per atom (MN_y_, y > 1) can significantly increase the volumetric energy densities of these substrates.^[^ ^70^ ^]^ However, this property is also the primary cause of high‐volume expansion.^[^ ^71^ ^]^ Additionally, the significant phase change in reconstitution reactions results in increased discharge‐charge voltage hysteresis and higher nucleation barriers compared to solid solution reactions.^[^ ^72^ ^]^ Yan et al. experimentally investigated the influence of 11 substrate materials with varying solubility in Li on nucleation overpotential.^[^ ^73^ ^]^ A clear overpotential was observed in the voltage profiles for the Li nucleation on materials that do not form alloys with Li (Cu and Ni) (Figure 6E) On the other hand, the Li nucleation overpotential was essentially zero for materials with definitive solubility in Li, such as Au, Ag, Zn, and Mg (Figure 6F). The authors conclude that the formation of a solid‐solution alloy during Li deposition acts as a buffer layer, being the main responsible for reducing the nucleation energy, thus reducing the overpotential.

The lithiation/sodiation/potassiation rate on alloying substrates can be influenced by the introduction of a diffusion pathway towards the metal, forming the alloy during cycling. The larger ionic sizes of Na and K can affect the migration barriers within the substrate structure, as demonstrated by DFT calculations in the work of Chou et al.^[^ ^74^ ^]^ A higher migration barrier was observed for a single Na atom, compared to Li, inside Si, Ge, and Sn substrate lattices. The addition of a sodium atom to the lattice reduced the Na migration barriers, suggesting that sodiation occurs faster at higher Na concentrations. A computational screening of Li nucleation on different intermetallic alloy surfaces was conducted in the work of Pande and Viswanathan.^[^ ^75^ ^]^ The authors employed DFT calculations to estimate the nucleation overpotential and Li diffusion barriers on alloy surfaces, using both a single atom and a full monolayer of lithium on alloy substrate surfaces as descriptors. Substrates where Li binds more strongly (lower nucleation overpotential) exhibited higher Li diffusion barriers. The authors identified a relationship between these properties and proposed substrates with optimized performance for uniform two‐dimensional growth of Li.

### 2.5 Dendrite Growth

Dendrite formations are one of the most common and plagued issues, that hindered the practical and scalable application of AMBs and ZEMBs.^[^ ^76^ ^]^ The continually growing dendrites can pierce through separators and cause internal short circuits, leading to the failures of batteries, and even catastrophic safety hazards, such as fires and explosions caused by overheating and thermal runaway.^[^ ^6b^ ^]^ A fundamental understanding of the mechanism of dendrite formation/ growth will facilitate innovative and effective solutions.

**Figure 7:**
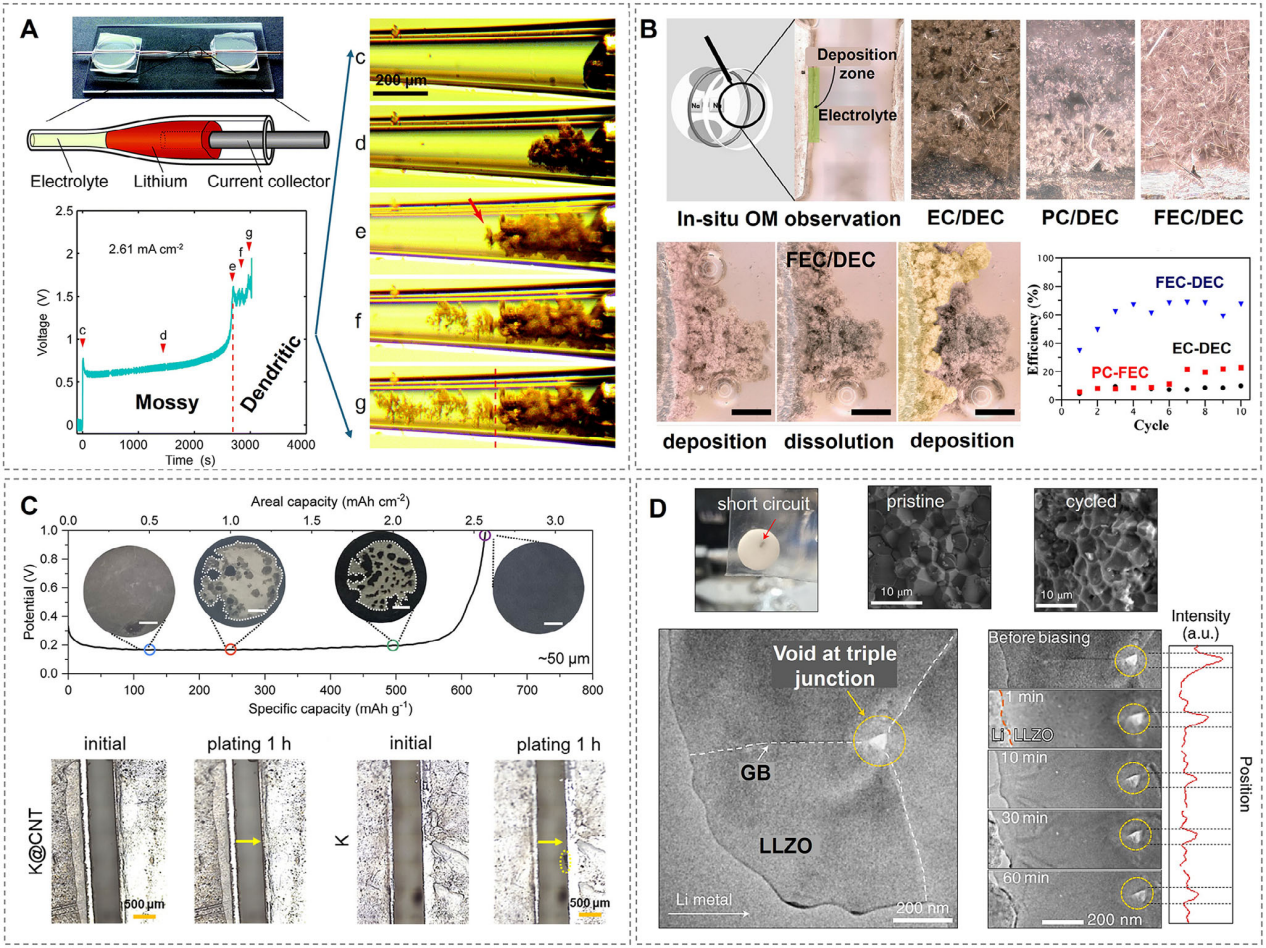
Morphological characterizations of alkali metal dendrites. A) The observation of Li dendrite growth in real‐time by a glass capillary cell. (Reproduced under the terms of the Creative Commons Attribution‐Non Commercial 3.0 Unported Licence.^[^ ^77^ ^]^ Copyright 2016, The Royal Society of Chemistry). B) Na electrodeposition in different electrolytes. (Reproduced with permission.^[^ ^78^ ^]^ Copyright 2017, American Chemical Society). C) K deposition behavior and characterization. (Reproduced with permission.^[^ ^79^ ^]^ Copyright 2024, American Chemical Society). D) Li dendrite formation in the grain boundaries. Reproduced with permission.^[^ ^80^ ^]^ Copyright 2021, The Author(s), under exclusive licence to Springer Nature Limited).

Over the past decade, significant progress has been made in understanding the nucleation and growth mechanisms of metal dendrite formation through experimental studies. For example, Bai et al. investigated the fundamental mechanisms behind Li dendrite growth in liquid electrolytes, utilizing a glass capillary cell to observe Li dendrite growth in real‐time (**Figure** **7**A).^[^ ^77^ ^]^ Their study identified two distinct dendrite growth modes: a mossy‐type Li dendrite with a root‐growing pattern under reaction‐limited conditions (low currents) and a dendritic‐type Li dendrite with a tip‐growing pattern when electrolyte diffusion became the limiting factor. Similarly, Rodriguez et al. reported that Na metal batteries also suffered from dendrite formation.^[^ ^78^ ^]^ Using a hermetically sealed optical cell, they observed Na deposition and compared Na growth morphologies in different electrolyte formulations (Figure 7B). Their key findings revealed that Na dendrites exhibited either a thin, needle‐like structure or a mossy‐like pattern. However, unlike Li dendrites, where clear factors determine different growth types, no significant evidence was found to distinguish Na dendrite formation mechanisms. For K metal batteries, dendrite formation remains a major challenge due to significant volume expansion and the pronounced “tip effect” during plating/stripping cycles, as shown in Figure 7C.^[^ ^79^ ^]^

Additionally, all‐solid‐state batteries, despite receiving increasing attention in recent years, continue to struggle with dendrite formation, particularly in Li metal anodes.^[^ ^80^ ^]^ Liu et al. summarized two primary dendrite formation mechanisms in LLZO.^[^ ^80^ ^]^ The first mechanism involves non‐uniform Li plating due to poor interfacial contact between the electrode and electrolyte (Figure 7D), which occurs rapidly and is commonly associated with early‐stage dendrite formation. The second mechanism occurs within the solid electrolyte grain boundaries, where Li ions are reduced, forming isolated filaments that gradually propagate through the electrolyte bulk. To further investigate Li dendrite propagation, Swamy et al. conducted experiments using single‐crystal LLZO, effectively eliminating the influence of grain boundaries.^[^ ^81^ ^]^ Their findings revealed that Li metal initiation led to cracks propagating through the electrolyte under high current densities. They concluded that the electric field played a dominant role in driving Li penetration. Similarly, Na dendrite formation has also been observed in solid‐state electrolytes. Jolly et al. reported that non‐uniform plating/stripping cycles resulted in continuous void formation, eventually leading to dendrite growth.^[^ ^82^ ^]^ The same group also observed similar dendrite penetration and void formation in K metal batteries under high current densities.^[^ ^83^ ^]^

Despite significant experimental progress in understanding dendrite formation, experimental techniques alone are insufficient to fully characterize the dynamic evolution of alkali metal dendrite growth mechanisms under various working conditions, such as electric fields, defect structures, and operating parameters. To further unravel these microscopic mechanisms, theoretical simulation models are essential. The following section introduces five common models, including the phase‐field model,^[^ ^84^ ^]^ the space‐charge model,^[^ ^85^ ^]^ the SEI‐induced model,^[^ ^7^, ^86^ ^]^ the heterogeneous nucleation model,^[^ ^60c^ ^]^ and the stress‐driven model.^[^ ^87^ ^]^ These models are constantly optimized and, to some extent, describe the dendrite formation and growth behavior under certain conditions. For instance, the space‐charge model is widely used to analyze the nucleation process based on Li⁺ diffusion behavior near the electrode. In this model, Li⁺ depletion creates a charge space, which contributes to dendrite formation. However, the formation and influence of the SEI are often underrepresented in the space‐charge model. In contrast, the SEI‐induced model is considered more effective in describing Li⁺ diffusion and deposition behavior at the electrode/electrolyte interface. Nevertheless, the SEI's composition and structure are highly complex, varying significantly with electrolyte type and undergoing dynamic changes during cycling. These complexities make reaching a unified conclusion about the SEI's functions challenging. It is widely believed that the nonuniform and porous structure of the SEI can result in uneven Li plating, while SEI fractures further exacerbate dendrite growth. Though boundary conditions and the applicable scope of various models for Li dendrite are well‐organized and discussed in some excellent reviews,^[^ ^88^ ^]^ the feasibility of applying these theories to Na/K alkali metal systems and comparative analysis is necessary.

**Figure 8:**
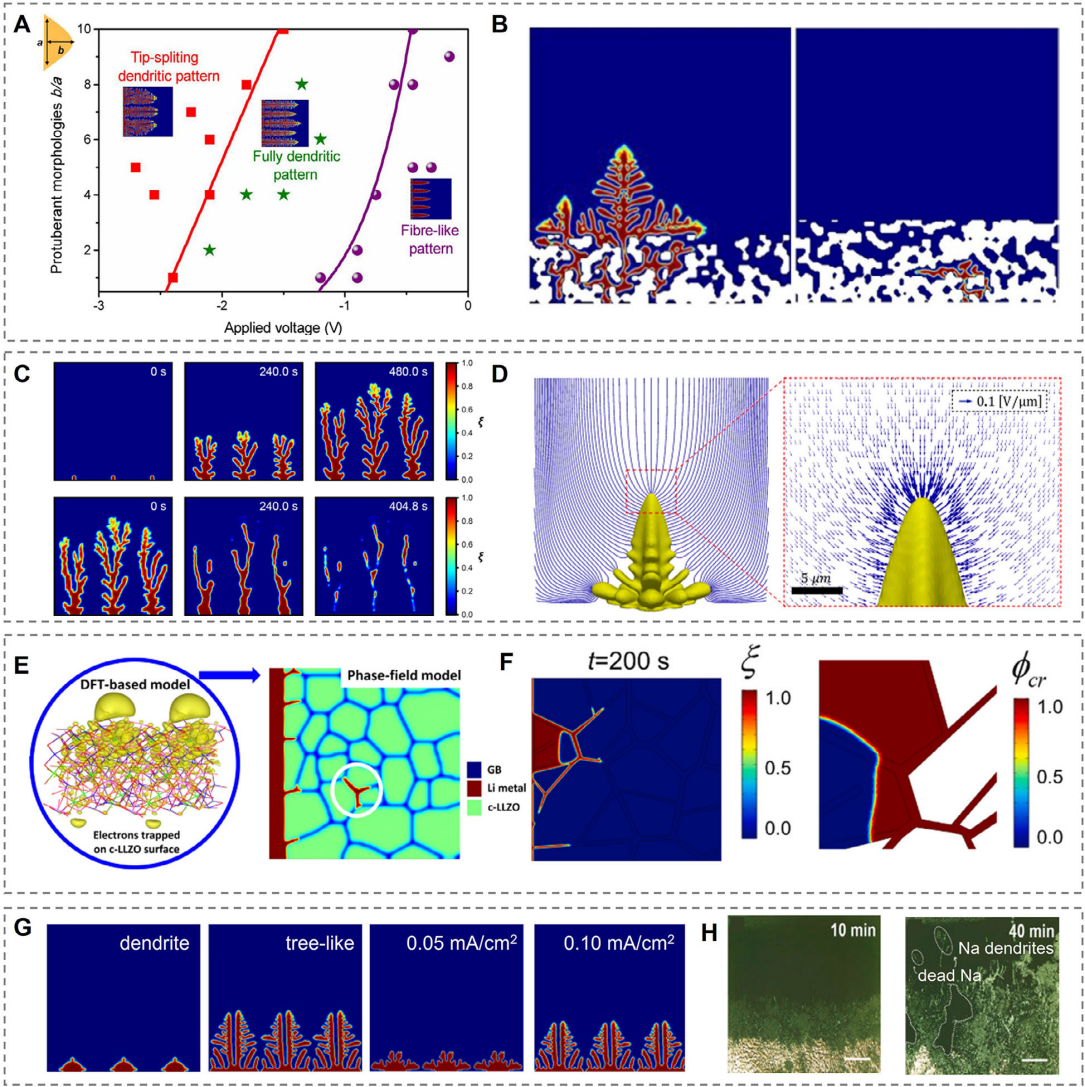
Dendrite morphology via phase‐field modeling. A) Morphological evolution of Li dendrites when varying applied voltages, illustrating tip‐splitting, fully dendritic, and fiber‐like patterns. (Reproduced with permission.^[^ ^84a^ ^]^ Copyright 2015, Elsevier B.V). B) Structural differences in the SEI layer, showcasing opened and closed configurations that influence Li growth behavior. (Reproduced with permission.^[^ ^84b^ ^]^ Copyright 2019, Elsevier B.V). C) Formation of dead Li during cycling, highlighting its impact on capacity loss and performance degradation. (Reproduced with permission.^[^ ^84c^ ^]^ Copyright 2022, Elsevier B.V). D) 3D representation of Li dendrite growth, emphasizing the complexity of dendritic structures in real‐world scenarios. (Reproduced with permission.^[^ ^84d^ ^]^ Copyright 2022, Elsevier B.V). E) The effects of surface‐trapped electrons by phase‐field simulation (Reproduced with permission.^[^ ^89^ ^]^ Copyright 2019, American Chemical Society). F) Phase‐field simulation results with pre‐defect: Li dendrite and crack area propagation. (Reproduced with permission.^[^ ^90^ ^]^ Copyright 2021, Elsevier B.V). G) The evolution of Na dendrite growth with different dendrite nuclei and varying current densities. (Reproduced with permission.^[^ ^91a^ ^]^ Copyright 2024, Elsevier B.V). H) In situ observation of Na dendrite growth. (Reproduced with permission.^[^ ^93^ ^]^ Copyright 2023, Elsevier Inc.).

The phase‐field model is a robust theoretical approach to simulate the microstructure of Li dendrite in liquid electrolyte batteries. The fundamental is to apply phase‐field order parameters to represent the transition between solid (Li dendrite) and liquid (electrolyte) phases. Chen et al. were the first to combine the standard phase field model (Gibbs free energy) and electrochemical overpotential equations, which were driven by electrostatic potential and ion concentrations.^[^ ^84a^ ^]^ This phase‐field model can predict the deposition/dendrite morphology in liquid electrolytes depending on various functions, such as current density, diffusion coefficients, nucleation radius, and interfacial energy. The authors concluded three Li dendrite patterns, including a tip‐splitting pattern, a fully dendritic pattern, and a fiber‐like pattern based on different voltages (**Figure** **8**A). Based on this standard phase‐field model platform, other researchers continued to develop the model for more complex scenarios. For example, Mu et al. considered the structure of the SEI layer, and they found that the SEI layer was beneficial in suppressing dendrite growth (Figure 8B).^[^ ^84b^ ^]^ The phase field model can also be used to describe the Li stripping procedures and investigate the dead Li formation (Figure 8C).^[^ ^84c^ ^]^ Specifically, the phase field model can reveal the relationship between the polarization curve and capacity loss peak. The phase field model was able to transfer from 2D to 3D. Arguello et al. used an open‐source finite element library to describe three‐dimensional Li dendrite, as shown in Figure 8D.^[^ ^84d^ ^]^ In the three‐dimensional simulations, the interactions between Li dendrites created a show to inhibit the branching growth, which is not possible to observe in a 2D model. There is also phase‐field modelling that presents key findings and novel contributions regarding the mechanism of Li dendrite in solidstate electrolytes (SSEs). Tian et al. developed a multiscale model combining phase‐field simulations and DFT calculations.^[^ ^89^ ^]^ Two cases were compared to identify the Li dendrite growth, which was with (real SSEs) and no excess (ideal SSEs) surface electron concentration, separately (Figure 8E). The results indicated that the dendrite growth was nonuniform and featured isolated Li‐metal nucleation in real SEs while it was continuous growth and following predictable paths in ideal SEs. Yuan et al. integrated electrochemical and defect mechanical forces in the phase‐field model to investigate dendrite and crack behaviors in SE.^[^ ^90^ ^]^ The novel point was to explore the effect of defects on Li dendrite and crack behaviors, as shown in Figure 8F. They claimed that the Li dendrites prefer to grow in grain boundaries and crack regions because of the reduced mechanical stiffness and increased electronic conductivity in those areas. The model also identified that crack‐related dendrite growth can be mitigated mechanically, but grain boundary dendrite growth is mostly driven by electromechanical forces.

The second model is the space‐charge model, which provides a theoretical framework to describe deposition behaviors. Ding et al. introduce a novel self‐healing electrostatic shield (SHES) mechanism to suppress lithium dendrite formation during metal deposition.^15^ This shield was able to prevent Li+ deposition on high‐field regions, especially for the dendrite tips. As a result, the Li deposition was redistributed to adjacent areas and created a smooth surface.

The next two are the SEI‐induced model and the heterogeneous nucleation model. The SEI‐induced model can describe the influence of SEI on the growth of Li dendrites. In 1979, Peled first introduced the SEI model to strengthen the understanding of alkali metal electrochemistry in nonaqueous battery systems.^[^ ^7^ ^]^ The importance of his work is to shift the perspective from treating alkali metal systems as simple charge transfer reactions to a complex SEI layered process, which is extremely important to high‐energy battery systems. Differently, the heterogeneous nucleation model focuses on understanding the thermodynamic and kinetic properties of Li electrodeposits.^[^ ^19^ ^]^ The study integrated the principles of electrochemistry with nucleation theory to present strategies for dendrite mitigation. It also emphasized the importance of several factors, such as the surface tension, overpotential, and substrate properties for critical stable electrodeposition conditions.

The final model is the stress‐driven model to investigate the Li dendrite. It should be noted that the stress‐driven model normally applies to organic electrolytes rather than inorganic types. In 1998, Yamaki et al. provided a new understanding of the effects of mechanical stress on Li dendrite structure.^20^ It claimed that stress was the key driving force to generate the whisker‐type dendrite formation. Wang et al. used a stress‐driven dendrite growth model to address the mechanism when they deposited Li on soft substrates.^21^ They found that hard substrates led to sharp and uneven dendritic growth due to stress concentration, while the soft substrates enabled more uniform deposition without sharp dendritic growth. Therefore, the stress‐relief approach might be another direction to suppress Li dendrite compared to normal techniques (e.g., electrolyte additives and SEI engineering).

It is important to confirm the feasibility of applying Li dendrite growth fundamentals to Na/K metals as previous research has made great progress for Li dendrite. For the phase field model, the input parameters are only associated with the physical properties of individual metals. Therefore, the equilibrium and kinetics equations still work without specific metals and the phase‐field model for alkali metals also confirms the feasibility.^[^ ^91^ ^]^ Space‐charge model is widely used in different metals without specific considerations.^[^ ^92^ ^]^ The SEI model is also valid for all alkali metals.^[^ ^8^ ^]^ The nucleation and growth of three alkali metals should follow the same fundamental thermodynamic principles, so the heterogeneous nucleation model for Li can be adaptive to Na/K metals with thermodynamic and electrochemical parameters adjustment. At the same time, there is no significant difference when applying the mechanical stress to the three alkali metals. For example, Gao et al. investigated the impact of current density on the evolution of Na dendrite morphology (Figure 8G). Their simulation results demonstrated that increasing the current density not only accelerates dendrite growth but also promotes the development of lateral branches. This behavior is qualitatively consistent with experimental observations reported by Ji et al. (Figure 8H).^[^ ^93^ ^]^

In conclusion, the technical route to apply Li dendrite models to Na and K metals is feasible. However, the dendrite challenges still exist in three alkali metal anodes. It is widely known that several factors influence the morphology of electrodeposited metal and cause the dendrite, such as current density, SEI layer, ion transport and mechanical properties of electrolyte, temperature, pressure, and tip radius of the protrusion.^[^ ^94^ ^]^ The common strategies to suppress the dendrite are novel anode design, new SEI design, artificial SEI layers, and solid‐state electrolytes. To date, most research focuses on the Li metal anode while Na/K is still at an early stage. It is urgently needed to bridge the gap between Li and Na/K metal anodes and compare the differences with their chemical properties, physical properties, electrochemical properties, and more unstable SEI layers.

## 3 Strategies for Stabilizing Anode

Although there is no universal solution for avoiding dendrite, some strategies based on theoretical model analysis and experimental observations show inhibitory or mitigated effects, including 1) designing uniform lipophilic sites/surfaces (e.g., seed) to guide the nucleation and deposition;^[^ ^73^ ^]^ 2) lowering the local current density by structural design (e.g., 3D structured scaffolds);^[^ ^95^ ^]^ 3) optimizing the SEI structure (e.g., electrolyte additives) to adjust Li^+^ desolvation, diffusion, and nucleation processes;^[^ ^96^ ^]^ 4) achieving beneficial surface's properties (e.g., crystal orientation, low roughness).^[^ ^97^ ^]^ In this section, we will discuss the basic model and theoretical understanding of the various strategies and highlight the advantages and disadvantages of each strategy, as well as the applicability to AMBs.

### 3.1 3D Structured Scaffolds

**Figure 9:**
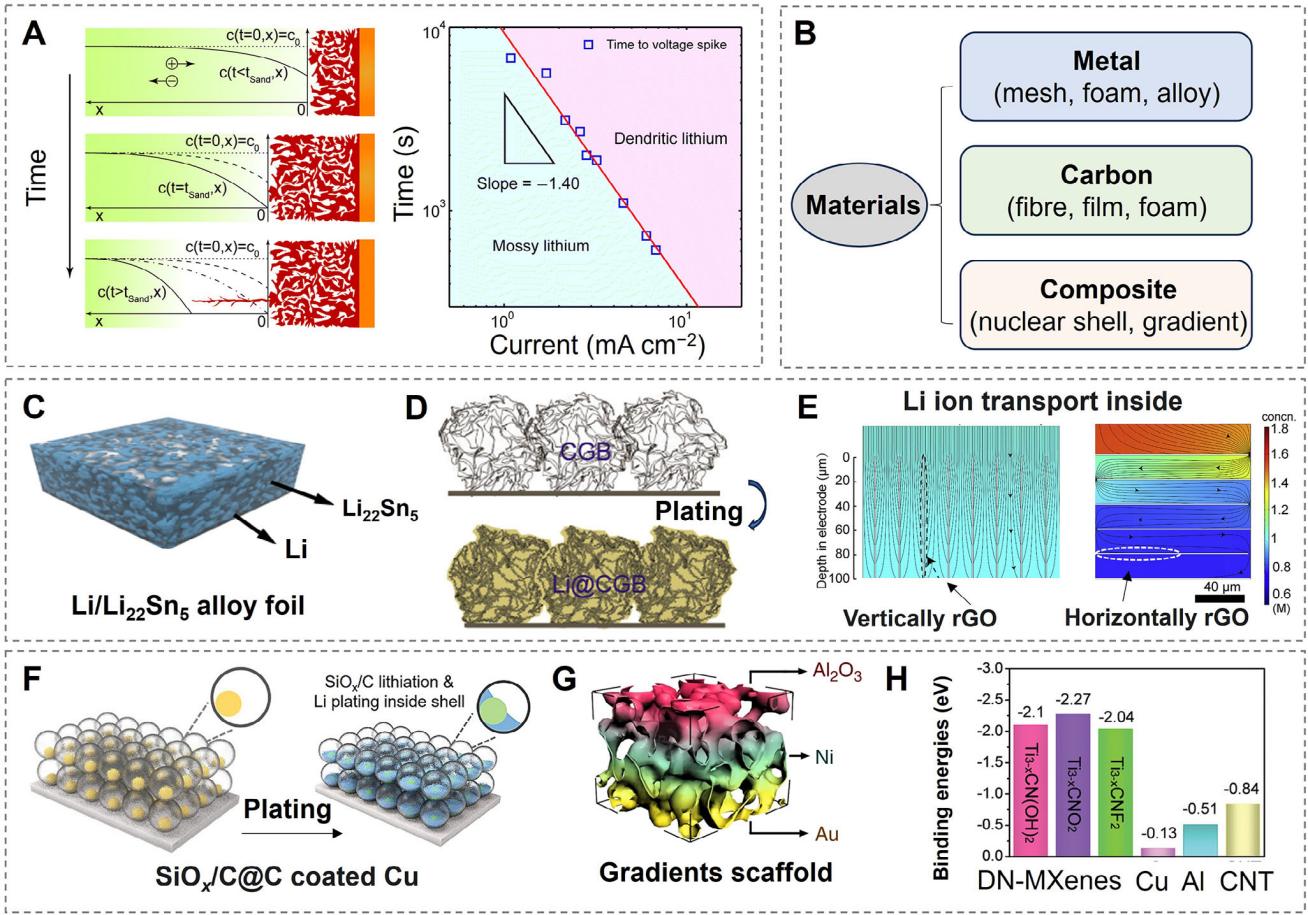
Metal plating on 3D structure scaffolds. A) Theoretical analysis of Li electrodeposition growth mechanisms under concentration polarization (left) and a log‐log plot showing experimental Sand's times for different current densities (right). (Reproduced under the terms of the Creative Commons Attribution‐Non Commercial 3.0 Unported Licence.^[^ ^77^ ^]^ Copyright 2016, The Royal Society of Chemistry). B) Overview of typical materials used in 3D scaffold structures. C–E) Schematic illustrations of representative 3D scaffolded structures. C) Li/Li₂₂Sn₅ nanocomposite foil showcasing enhanced nucleation and plating performance. (Reproduced with permission.^[^ ^104^ ^]^ Copyright 2020, Springer Nature Limited). D) Crumpled paper ball‐like graphene particles enable stable Li plating/stripping. (Reproduced with permission.^[^ ^109^ ^]^ Copyright 2020, Elsevier Inc.). E) Simulation of Li‐concentration distribution in VGA and HGA electrode configurations. (Reproduced with permission.^[^ ^110^ ^]^ Copyright 2020, Elsevier Inc.). F) The yolk–shell structure of SiOx/C@C featuring lithiophilicity gradients. (Reproduced with permission.^[^ ^111c^ ^]^ Copyright 2021, WILEY‐VCH Verlag GmbH & Co. KGaA, Weinheim). G) Conductivity and lithophilic gradient structures designed to optimize Li deposition and improve cycle stability. (Reproduced under the terms of the Creative Commons CC BY license.^[^ ^112^ ^]^ Copyright 2019, Springer Nature Limited). H) The calculated binding energies of K atoms with different scaffolds/collectors. (Reproduced with permission.^[^ ^61b^ ^]^ Copyright 2019, WILEY‐VCH Verlag GmbH & Co. KGaA, Weinheim).

3D‐structured scaffolds address dendrite‐related challenges by providing a conductive and porous framework that stabilizes the metal anode. The conductive nature of these scaffolds ensures efficient electron transport, reducing localized current density and mitigating the adverse effects of high local currents.^[^ ^98^ ^]^ The Sand's time model explains the relationship between current density distribution and dendrite formation. In dilute electrolyte solutions, cations are consumed at the electrode surface, creating a concentration gradient as ions fail to replenish the surface rapidly. Eventually, the cation concentration at the electrode surface drops to zero, defined as Sand's time (τ_s_). This depletion causes a breakdown in electrical neutrality, forming a space charge region that induces dendritic metal deposition. Sand's time is inversely proportional to the effective current density, J, as shown in the equation:^[^ ^99^ ^]^

(1) $$\tau_{s}=\frac{C_{0}e}{JD\left(u_{a}+\mu_{c}\right)}$$

here, D is the diffusion coefficient, e is the electronic charge, C_0_ is the initial electrolyte concentration, µ_a_ and µ_c_ are the transference numbers of anions and cations, respectively. Reducing current density extends Sand's time, delaying dendrite formation. Experimental measurements using in‐situ snapshots have validated this relationship, as shown in **Figure** **9**A, with current density versus time plotted on a logarithmic scale.^[^ ^77^ ^]^ The onset of dendrite formation depends on both current density and time, collectively termed “Sand's capacity”, which determines whether the morphology is mossy or dendritic.

Constructing a 3D scaffolded structure effectively enhances the specific surface area of the electrode while significantly reducing the local current density. This, in turn, decreases the ion consumption rate per unit area, mitigating concentration polarization and delaying the growth of dendrites. Moreover, metal deposition can be preferentially directed into the pores by tailoring the pore structure, buffering volume expansion and alleviating mechanical stress during cycling. Various types of 3D‐structured scaffolds have been developed for Li,^[^ ^100^ ^]^ Na,^[^ ^101^ ^]^ and K metal batteries,^[^ ^102^ ^]^ demonstrating significant improvements in cycling stability. Among these, metal alloys/foams, carbon hosts, and composite structures are the most extensively studied and hold great promise (Figure 9B). A comparative analysis of their key parameters and design principles provides valuable insights into their potential for practical applications.

3D alloy and metal foam scaffolds are highly valued for their mechanical robustness and low (Li/Na/K)‐phobic properties, which are crucial for uniform nucleation, maintaining anode integrity, and preventing issues such as cracking, collapse, or loss of structural support. Various Li‐M (M = Al, Mg, Zn, Sn, Ag, and Au) alloys have been extensively explored as 3D‐structured scaffold anodes, demonstrating significant reductions in Li nucleation polarization and improved cycling stability.^[^ ^95^, ^103^ ^]^ For example, a cross‐linked Li_22_Sn_5_ network host was fabricated using a calendaring and folding approach (Figure 9C).^[^ ^104^ ^]^ This structure exhibited a low overpotential of 20 mV at an extremely high current density and capacity of 30 mA cm⁻^2^ for 5 mAh cm⁻^2^. Notably, this simple calendaring method is compatible with existing electrode manufacturing processes and demonstrates excellent scalability. Unlike lithium, sodium and potassium metals have lower mechanical strength and a higher adhesive nature, leading to poor processability and structural stability. These deficiencies can be addressed using alloys. Similar to lithium, Na‐M (M = Au, Sn, Bi, Mg, and Sb) alloys have been investigated as sodiophilic hosts to achieve stable sodium metal anodes.^[^ ^101^ ^]^ In comparison to Li and Na, K alloys remain less studied, with only a few cases reported, such as K_3_Bi@K anodes.^[^ ^105^ ^]^ Further theoretical simulations and experimental studies are required to evaluate the feasibility of potassium alloys as anodes.

Metal foams offer similar benefits to alloys, such as mechanical robustness, but porosity is a critical factor that must be carefully optimized. Adequate porosity provides sufficient space for metal deposition while maintaining mechanical stability. However, excessive porosity can reduce the electrode's volumetric energy density, whereas insufficient porosity can lead to uneven metal plating. For instance, Yun et al. observed that the CE of commercial Cu foam, with pore diameters ranging from 100 to 400 µm, dropped below 90% after just 29 cycles.^[^ ^106^ ^]^ In contrast, 3D small porous Cu foam, with pore diameters of 0.2 to 2 µm, maintained a CE of 97% even after 250 cycles. Another critical challenge is the large nucleation overpotential caused by the low (Li/Na/K)‐phobic nature of Cu, which results in nonuniform metal deposition. While various strategies have been developed to enhance infiltrability and address these issues, the improvements are often confined to the small‐scale performance of individual cells. Factors such as scalability, large‐scale manufacturing, and cost‐effectiveness must also be addressed for broader applicability.

Compared with metal scaffolds, carbon‐based materials, including carbon paper, carbon cloth, carbon fiber film, graphene aerogels, carbon nanotubes (CNTs), and carbon foams, are widely utilized for their excellent chemical stability and low density.^[^ ^107^ ^]^ However, many commercial carbon materials (e.g., carbon cloth) suffer from poor metal wettability, which not only complicates the preparation of composite electrodes but also leads to various operational issues, such as uneven nucleation and high nucleation overpotentials. To address these challenges, surface chemistry plays a critical role in enhancing the functionality of 3D scaffolds. For example, commercial carbon cloth suffers from poor Li wettability, but functionalizing the carbon cloth with polar groups can improve its wettability and affinity for metal ions, thus promoting more uniform metal deposition.^[^ ^108^ ^]^ In addition to the modification of commercial carbon products, numerous carbon‐based 3D structures have been reported as scaffolds to stabilize metal anodes.^[^ ^107^ ^]^ For example, crumpled paper ball‐like graphene particles have been assembled into a scaffold that enables stable Li plating/stripping without dendrite formation, even at high Li loadings of up to 12 mA h cm⁻^2^ (Figure 9D).^[^ ^109^ ^]^ This excellent performance is attributed to the lithiophilic nature of the scaffold, which facilitates fast Li‐ion diffusion while accommodating volume fluctuations. Furthermore, the impact of tortuosity on Li plating behavior was systematically investigated by comparing reduced graphene oxide (rGO) hosts with different structural alignments, including vertically aligned (VGA), horizontally aligned (HGA), and randomly oriented configurations.^[^ ^110^ ^]^ The VGA structure, with low tortuosity (1.25), provides direct and continuous ion transport pathways, enabling uniform Li‐ion distribution and homogeneous Li deposition throughout the host. In contrast, the HGA structure, with significantly higher tortuosity (4.46), restricts ion diffusion, resulting in pronounced Li‐ion concentration gradients and elevated local current densities on the electrode surface. This uneven ion transport promotes preferential Li plating at the surface, increasing the risk of dendrite growth and capacity degradation. Finite element simulations using COMSOL Multiphysics further confirmed these trends (Figure 9E), showing only a 2.7% Li‐ion concentration drop and a minimal 3.6% current density difference across the VGA structure, while the HGA exhibited a 43.2% ion concentration drop and a 283% increase in surface current density. These findings demonstrate that minimizing tortuosity through structural alignment effectively mitigates ion transport limitations and current inhomogeneities, which are critical for achieving stable, dendrite‐free Li plating/stripping. This work provides valuable insights into the rational design of advanced scaffolds for high‐performance metal anodes.

Composite scaffolds, including core–shell structures, polymer–ceramic composites, and gradient architectures, are emerging as promising hosts for metal anodes. By integrating multiple components with complementary properties, these scaffolds can fulfill specific functions to enhance electrochemical performance.^[^ ^111^ ^]^ For instance, the introduction of (Li/Na/K)‐phobic sites within the scaffold can effectively regulate nucleation behavior and promote uniform metal plating. He et al. designed an electroactive yolk–shell structure of SiOx/C@C featuring deliberately engineered lithiophilicity gradients (Figure 9F).^[^ ^111c^ ^]^ In this design, the SiOx/C core exhibits higher lithiophilicity than the doped carbon shell, guiding Li nucleation and growth to initiate from the SiOx/C core and subsequently extend into the surrounding interparticle spaces. This controlled plating sequence effectively accommodates volume expansion and ensures uniform Li deposition and stripping, thereby improving the structural stability and cycling performance of the anode. The other is to build gradient skeletons. For example, a highly porous nickel scaffold with a top Al_2_O_3_ coating and a bottom Au coating forms a lithiophilicity gradient (Figure 9G), showing a high CE of ≈98.1% after 500 cycles at 3.5 Ma h cm^−2^/2 mA cm^−2^.^[^ ^112^ ^]^ Although these ingenious designs show excellent performance improvements, achieving large‐scale preparation is still a challenge. Similarly, the strategy of constructing gradient‐distributed structures is frequently employed to optimize Na or K plating. For example, Wang et al. designed a freestanding scaffold composed of CNTs and defect‐rich nitrogen‐containing MXene (DN‐MXene) (Figure 9H).^[^ ^61b^ ^]^ In comparison to Cu foil (−0.13 eV), Au foil (−0.51 eV), and CNT (−0.84 eV) scaffolds, which exhibit relatively lower binding energies with K atoms, the DN‐MXene with different terminal functional groups showed binding energies of 2.27, 2.10, and 2.04 eV for Cu, Al, and CNT, respectively. Thanks to its high potassium‐philicity, the DN‐MXene sheets act as nucleation sites, lowering the nucleation barrier and promoting uniform potassium nucleation and plating.

Despite these advancements, several challenges remain in the development and implementation of 3D scaffolds. A key concern is the trade‐off between energy density and scaffold mass, as the inclusion of a scaffold increases the electrode's weight and volume, potentially reducing its overall energy density. Therefore, it is a priority to develop lightweight, highly porous scaffolds that maintain their functionality. Additionally, scalability and cost are significant obstacles. Although advanced fabrication techniques, such as freeze‐drying,^[^ ^113^ ^]^ chemical vapor deposition,^[^ ^114^ ^]^ and 3D printing,^[^ ^115^ ^]^ can produce high‐performance scaffolds, they are often expensive and difficult to scale. Furthermore, the scaffold's chemical compatibility with reactive metals and electrolytes is crucial for ensuring long‐term stability.

### 3.2 SEI Design Through Electrolyte Optimization

In the pursuit of enhancing AMB's performance, the development of a protective barrier at the anode/electrolyte interface is paramount. This barrier, known as the solid electrolyte interphase (SEI), must inhibit electron flow while facilitating ion movement, thereby safeguarding the battery against adverse (electro)chemical reactions and the proliferation of dendrites. The SEI is a composite of organic and inorganic compounds, formed through the (electro)chemical degradation of the electrolyte on the metal anode, underscores a straightforward relationship between the electrolyte's composition and the SEI's properties.

In the realm of electrolyte engineering for SEI design, the focus is on carefully screening and combining solvents, salts, and additives to meet the diverse needs of ion transport and SEI formation. Achieving high ionic conductivity is essential for the efficient movement of ions and is a key target in electrolyte formulation.^[^ ^116^ ^]^ The electrolyte must also facilitate a high transference number for the target cation, such as Li^+^, Na^+^, and K^+^, to reduce concentration polarization and ensure smooth ion flow.^[^ ^117^ ^]^ The solvation shell that surrounds the ions significantly impacts ion transport properties.^[^ ^118^ ^]^ The electrolyte must provide a favorable solvation environment that boosts ionic mobility and lowers the energy barrier for ion movement.^[^ ^119^ ^]^ Furthermore, the physical properties of the electrolyte, including viscosity and flow characteristics, not only play a role in determining ionic conductivity and wetting behavior, but also affect practical aspects of battery production, such as the ease of filling and assembly.^[^ ^120^ ^]^ Maintaining thermal stability throughout the operational temperature range is critical to avoiding thermal decomposition, maintaining ion transport, and keeping the SEI intact, thus ensuring both the longevity and safety of the battery system.^[^ ^121^ ^]^

Besides the considerations in ion transport, the optimization of SEI formation necessitates a harmonious balance of chemical, physical, and electrochemical attributes. The stability of the electrolyte during electrode redox reactions and its compatibility with the metal anode are of utmost importance, necessitating a broad electrochemical stability window to mitigate decomposition and the formation of an unstable SEI, which could compromise battery performance.^[^ ^122^ ^]^ Tailoring the SEI's compositions are pivotal to achieving a uniform layer with the desired attributes, such as high ionic conductivity and robust mechanical properties.^[^ ^123^ ^]^ The durability and flexibility of the SEI are critical to enduring the mechanical stresses from massive volume changes of alkali metal anodes during cycling without cracking or peeling.^[^ ^124^ ^]^

**Figure 10:**
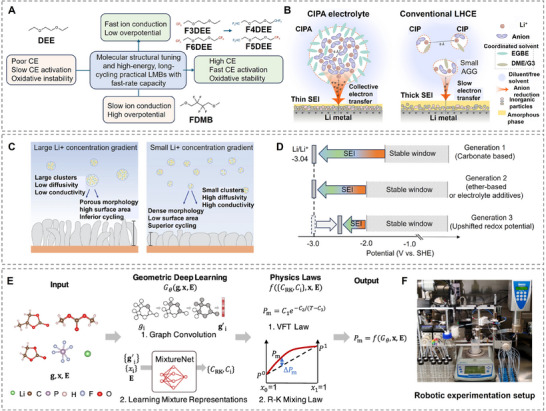
SEI design through electrolyte optimization. A) Development directions for high‐performance electrolytes, focusing on fast ion conduction, low overpotential, high Li metal deposition efficiency, rapid CE activation, and enhanced oxidative stability. (Reproduced with permission.^[^ ^130^ ^]^ Copyright 2022, Springer Nature Limited). B) Schematics illustrating the solvation structures and interfacial reaction mechanisms of the CIPA electrolyte compared to conventional LHCEs, highlighting the differences in stability and performance. (Reproduced with permission.^[^ ^135^ ^]^ Copyright 2022, Springer Nature Limited). C) High‐entropy electrolytes (HEEs) with smaller ion clusters demonstrating higher diffusivity and conductivity, resulting in reduced Li^+^ concentration gradients and denser Li deposition morphologies during high‐rate cycling. The schematic compares the performance of LEE (left) and HEE (right) configurations. (Reproduced with permission.^[^ ^136^ ^]^ Copyright 2023, Springer Nature Limited). D) Evolution of electrolyte concepts aiming to broaden the stable potential window for LMBs, showing milestones in electrolyte innovation. (Reproduced with permission.^[^ ^137^ ^]^ Copyright 2022, Springer Nature Limited). E,F) The differentiable, predictive model architecture and robotic experiment for electrolytes. (Reproduced with permission.^[^ ^116^ ^]^ Copyright 2024, Springer Nature Limited).

To refine the electrolyte formulation for optimal ion transport and SEI formation, the selection of the solvent plays a pivotal role in dissolving salts, determining the electrochemical stability window, enhancing ionic conductivity, and promoting the formation of the SEI. The ideal solvent should feature high boiling points, minimal volatility, and high dielectric constants. A solvent with a high dielectric constant can retain anions close to electrode surface, facilitating anion reduction for SEI formation.^[^ ^125^ ^]^ Salt choice should focus on those that serve as precursors to a robust SEI. The anion of salt can greatly influence SEI formation, as anions with low LUMO contribute to a more protective and resilient SEI due to the preferential decomposition of anions compared to the solvent.^[^ ^126^ ^]^ Additives are also influential in enhancing SEI stability and ion transport properties, thus improving battery performance. Fluorinated solvents, salts, or additives are particularly effective.^[^ ^127^ ^]^ Electron‐withdrawing inductive effect of fluorine atoms helps to stabilize the chemical structure of the solvents, salts, or additives, enhancing chemical stability.^[^ ^128^ ^]^ Fluorination also leads to higher reduction potential and promotes the decomposition of fluorinated solvents, salts, or additives on alkali metal anodes, thereby forming fluorine‐rich SEI, which can boost its thermal, mechanical, and electrochemical stability.^[^ ^129^ ^]^ Furthermore, the utilization of fluorinated solvents can significantly enhance ion transport by establishing a more conductive medium for alkali metal ions, a key factor for optimal battery performance. The precise locations and proportions of fluorination play a pivotal role in this process. Research conducted by Bao's group (**Figure** **10**A) has demonstrated that a partially fluorinated, locally polar –CHF_2_ group incorporated into 1,2‐diethoxyethane (DEE) yields superior ionic conductivity compared to a fully fluorinated –CF_3_ group. This leads to a reduction in overpotential and an acceleration in activation, thereby improving the overall efficiency and stability.^[^ ^130^ ^]^ In liquid electrolytes, the fluorine sources are typically limited to either negatively charged anions or fluorinated solvent molecules. However, Zhang and Xu et al. expanded this scope by synthesizing an ionic liquid (PMpyr_f_FSI) featuring a fluorinated cation, 1‐methyl‐1‐propyl‐3‐fluoropyrrolidinium, paired with a fluorinated anion, FSI^−^.^[^ ^131^ ^]^

The solvation of cations by solvent molecules plays a pivotal role in determining the characteristics of the SEI. As the solute concentration rises within an electrolyte, the types of ion pairs, with the anion serving as the reference point for counting the number of coordinating cations, undergoes a transformation from solvent‐separated ion pairs (SSIPs) to contact ion pairs (CIPs) and ultimately to aggregates (AGGs). HCEs are capable of forming more densely packed ion clusters, such as CIPs and AGGs, which in turn reduce the availability of free solvents.^[^ ^96^ ^]^ This can broaden the electrochemical stability window and lead to the formation of a less soluble, more stable SEI. However, HCEs may also present challenges, including reduced cost‐effectiveness, increased viscosity, and decreased tolerance to low temperatures.^[^ ^132^ ^]^ To address these issues, LHCEs have been developed. LHCEs are produced by diluting HCEs with a non‐solvating solvent that preserves the solvation structure of HCEs, effectively mitigating the drawbacks associated with HCEs.^[^ ^133^ ^]^ This strategy allows for the development of a robust SEI while maintaining a lower overall electrolyte concentration and minimizing viscosity, thus optimizing both performance and practicality. Interestingly, the role of a diluent in promoting LHCE formation is not limited to liquid solvents; salts can also serve this purpose. Manthiram's group has demonstrated that in a 1.1 M NaFSI‐trimethyl phosphate (TMP) electrolyte, the addition of 0.3 M NaNO_3_ can nudge the TMP out of the primary solvation structure due to the strong interactions between NaNO_3_ and TMP. This disruption results in the formation of AGGs incorporating NO_3_^−^, thereby reducing the amount of salt needed to create an LHCE.^[^ ^134^ ^]^

Recently Xu et al. have demonstrated that larger solvation clustering structures can support the operation of a 500 Wh kg^−1^ Li‐metal pouch cell for up to 130 cycles.^[^ ^135^ ^]^ They achieved this by creating a more compact ion‐pair electrolyte, composed of 2 M lithium bis(fluorosulfonyl)imide (LiFSI) in a 1:1 volume mixture of ethylene glycol di‐n‐butyl ether (EGBE) and 1,1,2,2‐tetrafluoroethyl 2,2,3,3‐tetrafluoropropyl ether (TTE). This electrolyte forms large aggregates (3–4 nm in size) with tightly packed ion pairs, leading to shorter Li^+^–Li^+^ distances of ≈6 Å. In contrast, conventional LHCEs that use regular ethers like diglyme (G2) or dimethoxyethane (DME) typically feature smaller aggregates (≈1 nm) and larger Li^+^–Li^+^ distances (≈8 Å). As shown in Figure 10B, the dense ion pair packing and large aggregates give rise to a collective electron transfer mechanism that facilitates the rapid reduction kinetics of the FSI^−^ anions. This unique interfacial reaction mechanism results in the formation of a stable SEI with a high inorganic content and an average thickness of ≈6.2 nm, which is crucial for the overall stability. Meanwhile, Cui et al. have shown that high‐entropy electrolytes (HEEs) with smaller ion clusters can enhance ionic conductivity and decrease concentration gradients, leading to more uniform Li deposition at high‐rate charging (Figure 10C).^[^ ^136^ ^]^ From these findings, we infer that there may be an optimal size for ion clusters in LHCEs that balances ion transport and SEI‐forming ability for ideal battery performance.

Notably, the solvation structure of Li^+^ ions also has a profound impact on the redox potential of the Li^+^/Li redox couple. As shown in Figure 10D, Yamada et al. have shown that the Li metal electrode's potential can increase by over 0.6 V when using an electrolyte composed of 1.5 m LiFSI dissolved in dimethoxymethane (DMM).^[^ ^137^ ^]^ This enhancement is attributed to the formation of ion clusters, such as CIPs and AGGs, as opposed to the predominant SSIPs found in electrolytes with conventional ethers like G2 or DME. The elevated electrode potential diminishes the reductive capacity of Li, which in turn results in less electrolyte decomposition and manifests as higher CE.^[^ ^108^ ^]^

Except for considerations in ion transport and SEI formation, the electrolyte must possess non‐flammable and non‐toxic properties to enhance the safety of batteries, particularly in applications where the battery is in close contact with consumers or in environments where safety is paramount.^[^ ^138^ ^]^ The components of the electrolyte should be affordable and readily obtainable to support the scalability and economic feasibility of battery technology. Additionally, the electrolyte should be environmentally benign, minimizing its impact throughout the production, usage, and disposal phases. Compliance with relevant regulations and standards is essential, especially for batteries employed in vehicles and consumer electronics. Safety, cycle life longevity, environmental friendliness, and cost‐effectiveness are all critical considerations, emphasizing the necessity for sustainable electrolyte materials. Addressing these complex challenges necessitates a comprehensive strategy that harmoniously balances stability, conductivity, compatibility, safety, and environmental factors. Further details of electrolyte design for metal anodes are available from previous review articles.^[^ ^96^, ^132^, ^139^ ^]^

While the central requirements for electrolytes and SEI are consistent for AMBs, the distinct characteristics of Li, Na, and K necessitate tailored designs. The larger size and lower charge density of Na^+^ and K^+^ ions compared to Li^+^ ions significantly influence the solvation structure, ionic transport properties, and interactions with electrode materials and electrolytes.^[^ ^76^, ^140^ ^]^ The diverse electrochemical stability windows of Li, Na, and K electrolytes underscore the inappropriateness of a one‐size‐fits‐all approach to electrolyte composition.^[^ ^141^ ^]^ The distinct mobility and transport properties of these cations, due to varying solvation capabilities and stokes radius, may necessitate the use of different solvents or additives to optimize battery performance.^[^ ^67^, ^142^ ^]^ The mechanisms underlying the formation of the SEI can differ considerably. Materials that effectively stabilize interfaces with Li may not necessarily provide the same benefits for Na or K systems. As a result, the principles established for LMBs may not be directly applicable to Na or K‐based batteries. For instance, incorporating 5% (by volume) fluoroethylene carbonate (FEC) as an electrolyte additive can lead to the development of an inorganic‐rich SEI that improves performance in LMBs.^[^ ^143^ ^]^ In contrast, the same concentration of FEC in PMBs can result in increased impedance and premature battery failure, stemming from the formation of a less conductive SEI layer.^[^ ^144^ ^]^

Furthermore, LMBs necessitate SEIs with a fine structure to manage the small size and high mobility of Li^+^ ions, facilitating their efficient passage through the SEI while preventing short circuits. In contrast, SMBs and PMBs may require SEIs with larger nanopores or alternative structural configurations to accommodate their larger ions without obstructing ion transport. The reactivity of each metal with the electrolyte also differs, necessitating SEI layers tailored to their individual electrolyte systems. For Li, the SEI must be highly stable, while for the more reactive Na and K, SEIs need to be more durable to withstand their aggressive interactions. Given that Na and K are more prone to dendrite formation, SEI designs for these metals must actively inhibit dendrite growth. This can be accomplished by increasing the SEI's mechanical strength or incorporating chemicals that impede the initiation and spread of dendrites. All in all, the disparities in ionic size, solvation ability, reactivity, electrolyte compatibility, and dendrite formation among Li, Na, and K metal batteries contribute to their unique attributes. These distinctions require tailored design approaches for each case to maximize both performance and safety.

The design and optimization of electrolytes remain a significant challenge due to their complex chemistry and the high cost of experimental screening.^[^ ^145^ ^]^ For future electrolyte development, various simulation methods can be combined with experimental approaches to guide electrolyte design. For example, physics‐based modeling techniques, such as MD, can provide insights into the underlying interactions and dynamic evolution within complex mixture systems.^[^ ^146^ ^]^ Additionally, mixture physics can be described using empirical functional relationships. Dynamic behavior and thermodynamics, for instance, are often modeled using the Arrhenius equation and Redlich–Kister (R‐K) polynomials, respectively, offering intrinsic physical insights.^[^ ^147^ ^]^ Moreover, emerging data‐driven methods, such as deep learning, can further enhance the predictive modeling of electrolyte mixtures. For example, Zhu et al. integrated geometric deep learning (GDL) with a robotic experimentation setup (Clio), demonstrating a differentiable optimization approach for battery electrolyte mixtures. Their work improved model accuracy and efficiency in electrolyte modeling and optimization, enabling the differentiable optimization of battery electrolyte properties (Figure 10E,F).^[^ ^116^ ^]^

### 3.3 Artificial SEI Design

**Figure 11:**
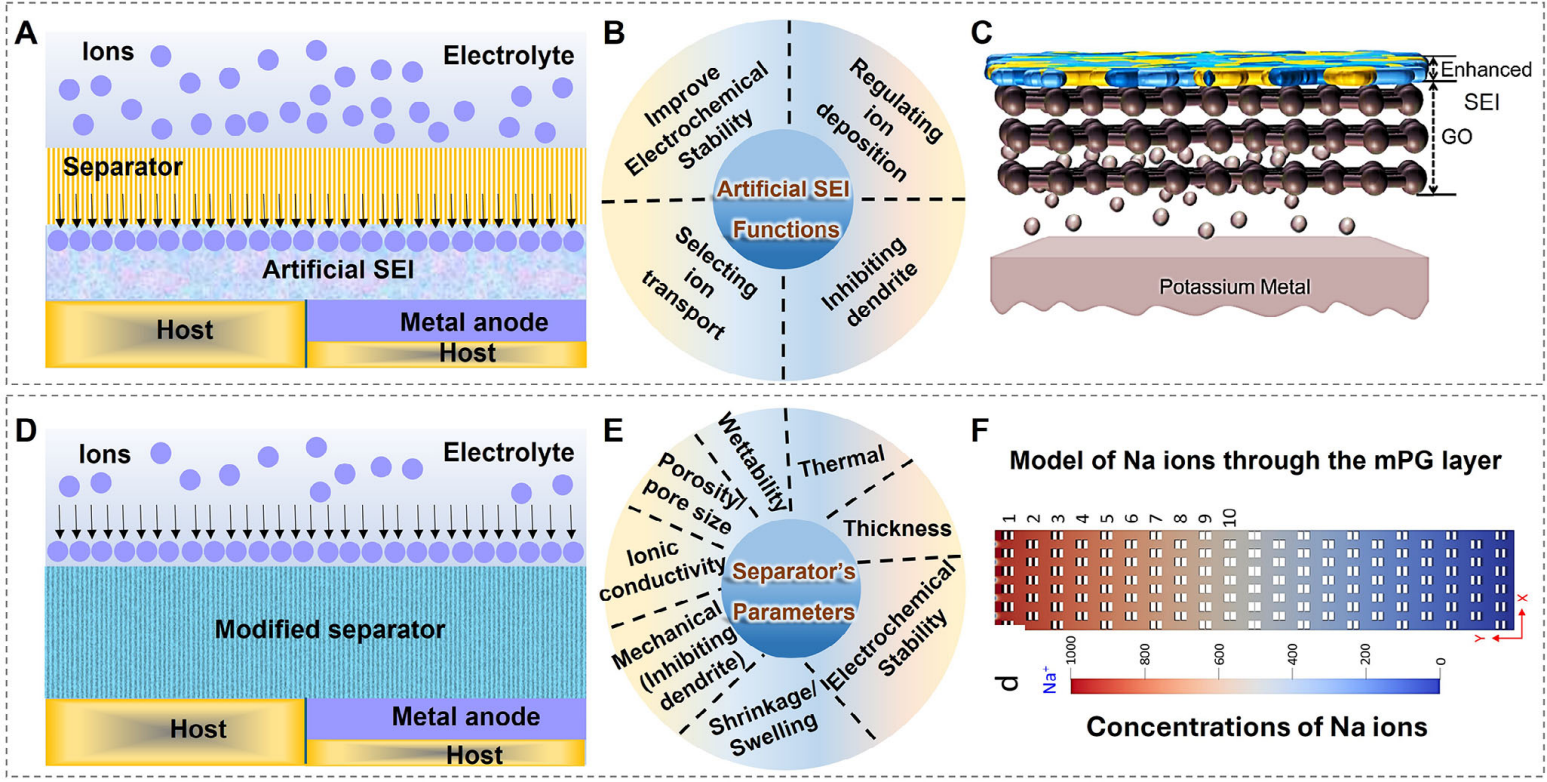
Artificial SEI construction and separator modifications. A) Schematic illustrations of artificial SEI construction in anode‐less and anode‐free configurations, demonstrating the effect of stabilizing the interface and mitigating side reactions. B) Summary of the functions provided by artificial SEI, including enhancing ion transport, reducing dendrite growth, and improving cycling stability. C) Schematic illustrations of the metal electrode skin structure and surface. (Reproduced under the terms of the Creative Commons CC BY license.^[^ ^162^ ^]^ Copyright 2023, The Author(s), under exclusive licence to Springer Nature Limited.) D) Schematic illustrations of separator modifications in anode‐less and anode‐free configurations, showcasing the effect of improving ionic conductivity, mechanical strength, and wettability. E) Summary of key parameters to modify separators, focusing on properties such as porosity, thermal stability, and electrolyte compatibility. F) Model diagram of Na ions through mPG layer (Inset: the color represents different concentrations of Na ions). (Reproduced under the terms of the Creative Commons CC BY license.^[^ ^180^ ^]^ Copyright 2021, The Author(s), under exclusive licence to Springer Nature Limited).

The SEI is a thin layer that forms on the surface of metal anodes in batteries, acting as a barrier between the anode and electrolyte. While crucial for preventing direct contact and undesirable reactions, the instability of the native SEI can lead to poor battery performance.^[^ ^93^ ^]^ Additionally, its inherent inhomogeneity causes uneven ionic flux and the native SEI dissolution gives rise to continuous Li corrosion, aggravating dendritic deposition and compromising battery longevity.^[^ ^148^ ^]^ To address these issues, researchers have focused on developing artificial SEIs for anode‐free and anode‐less battery structures (**Figure** **11**A), which are engineered coatings designed to improve battery operation.

Key attributes of an effective artificial SEI include balancing high ionic conductivity, allowing ions to pass without hindrance, and electronic insulation to prevent unwanted reactions.^[^ ^149^ ^]^ Its composition must be carefully designed to achieve both without compromising performance. Since the artificial SEI layer interacts with both the anode and the electrolyte, it must be sufficiently stable to maintain its structural integrity over time, preventing detrimental reactions that could degrade the layer. For example, incorporating lithiophilic, nitrogen‐rich polyethyleneimine (PEI) not only enhances Li⁺ solvation and regulates ion flux but also minimizes undesirable electrolyte swelling and solvent decomposition. This is achieved through its solvent‐phobic hexyl groups, which reduce electrolyte solvent affinity.^[^ ^150^ ^]^ Additionally, constructing a single‐ion conducting polymer is highly desirable, as it facilitates fast ion transport while preventing unwanted ion migration and side reactions.^[^ ^151^ ^]^ However, caution is needed, as exposure to electrolytes may compromise the polymer's single‐ion conductivity.^[^ ^152^ ^]^ This stability, combined with ionic conductivity, allows the artificial SEI to act as an exogenously introduced physical barrier while enabling ion transport, ensuring durability and enhanced battery performance. The artificial SEI must also withstand the expansion and contraction of the metal anode during charging and discharging cycles.^[^ ^153^ ^]^ High mechanical strength and flexibility ensure it remains intact, avoiding cracks that could impair battery functionality.^[^ ^154^ ^]^ Its thickness must be optimized to provide adequate protection while maintaining efficient ion transport, a cornerstone of battery efficiency. Additionally, favorable wettability with the electrolyte and strong adhesion to the electrode are critical.^[^ ^155^ ^]^ A well‐bonded artificial SEI with intimate electrolyte coverage ensures consistent performance and uniform deposition over the battery's operational life.^[^ ^156^ ^]^ The requirements of an effective artificial SEI are summarized in Figure 11B.

Fabricating artificial SEIs involves a range of advanced techniques, extensively documented in previous review articles.^[^ ^157^ ^]^ In essence, fundamental methods include physical or chemical vapor deposition^[^ ^158^ ^]^ and solution‐based processes like dip‐coating and spin‐coating,^[^ ^159^ ^]^ which create uniform, precise layers. For example, a dense Li_4_Ti_5_O_12_ (LTO) coating on copper foil induces a localized increase in Li^+^ concentration near the copper surface due to the formation of lithium‐rich LTO through Li^+^ intercalation. This Li^+^ concentration gradient opposes the ion concentration gradient in the electrolyte near the Li anode, thereby alleviating concentration polarization. As a result, it facilitates uniform lithium plating and stripping, particularly at high current densities.^[^ ^160^ ^]^ Besides, layer‐by‐layer assembly involves sequential deposition of materials to optimize properties at varying depths.^[^ ^161^ ^]^ For example, recent work by Lu et al. has introduced the concept of a skin‐like biomimetic protection layer to tackle the challenges associated with K metal anodes (Figure 11C).^[^ ^162^ ^]^ They achieved this by constructing a fluorine‐doped graphene oxide (F‐GO) protection layer on K metal foil using the Langmuir–Blodgett method. This strategy significantly enhances the flatness of the electrode surface, resulting in a uniform interfacial electric field that mitigates the “tip effect”. The “tip effect” arises when the flux of K^+^ ions is more concentrated at the tips of rough K metal, leading to uneven deposition and dendrite formation. The substantial surface area of the F‐GO layer facilitates the deposition of K metal into its interior spaces, which effectively mitigates the volume expansion of the interface, preserving its structural integrity. Moreover, the F‐GO layer not only improves the wettability of the electrolyte but also provides fluorine resources through carbon‐fluorine bond cleavage on the K metal surface. This process creates a fluoride‐rich SEI, which strengthens the mechanical properties of the SEI. SEM images and in‐situ optical microscopy observations of the K metal surface after cycling reveal a marked decrease in dendrite formation when the F‐GO‐modified surfaces are utilized.

In situ techniques create artificial SEIs through electrolyte reactions tailored to battery needs.^[^ ^163^ ^]^ Other innovative methods include sol‐gel processes,^[^ ^164^ ^]^ hydrothermal synthesis,^[^ ^165^ ^]^ and nanotechnology.^[^ ^166^ ^]^ Nanoscale materials like graphene or nanocomposites provide enhanced properties due to their high surface area and unique functionalities. Incorporating inorganic fillers in polymer matrices improves stability,^[^ ^167^ ^]^ while advanced techniques such as atomic layer deposition (ALD),^[^ ^168^ ^]^ molecular layer deposition (MLD),^[^ ^169^ ^]^ and plasma treatments allow precise control over artificial SEI attributes.^[^ ^170^ ^]^ For example, Al_2_O_3_–alucone alloy interfaces were fabricated on Li‐ and Na‐metal anodes using the ALD/MLD process, both demonstrating significant improvements in electrochemical performance in carbonate‐based electrolytes. However, the optimized alloy structures and interface thicknesses differ significantly between Li‐metal anodes (2ALD‐2MLD‐25) and Na‐metal anodes (1ALD‐1MLD‐10), with Na requiring a much thinner interface layer. This difference was attributed to the distinct chemical, electrochemical, and mechanical properties of Li and Na.^[^ ^171^ ^]^

### 3.4 Separator Modification

The separator is a critical battery component, serving as a physical barrier between the anode and cathode to prevent short circuits while regulating ion flux.^[^ ^172^ ^]^ The role of the separator is depicted in Figure 11D. Key considerations for designing separators include pore structure (size, arrangement, porosity, and tortuosity), which controls ion flux and uniform deposition, directly influencing ion transport rates. Material properties like chemical stability, erosion resistance, and mechanical strength ensure long‐term functionality and prevent decomposition under repeated cycling stresses. Wettability and electrolyte compatibility are essential for consistent ion flow and to prevent adverse reactions. Thickness affects strength and ion diffusion, while thermal conductivity aids in heat dissipation, critical for thermal management. Separators must also remain stable under operating conditions and block ion transport at extreme temperatures to prevent thermal runaway. Figure 11E summarizes the key considerations for separators in AMBs.

The separator interacts with key battery components such as the electrolyte and the SEI, influencing performance and safety. Separator pore structure and chemical composition play important roles in electrolyte behavior, affecting ion transport and battery impedance.^[^ ^173^ ^]^ The homogeneity of ion flux positively affects the long‐term cycle life of batteries. Aligned pores and low tortuosity enable rapid and even ion flux that uniformizes metal deposition.^[^ ^174^ ^]^ The optimally designed chemical composition of the separator enhances cation mobility by influencing the solvation shell of metal ions.^[^ ^175^ ^]^ Incorporating anion‐adsorbing sites immobilizes anions, improving cation efficiency, transference numbers, and the exchange current density while promoting wettability for fast‐charging capabilities.^[^ ^176^ ^]^

Computational methods, including DFT calculations, ab initio molecular dynamics simulations, and phase‐field simulations, provide deeper insights into separator‐electrolyte interactions. For instance, incorporating a separator with an antistatic agent, metal‐organic frameworks (MOFs), or covalent organic frameworks (COFs) facilitates PF_6_^−^ absorption from the electrolyte, promoting uniform Li deposition.^[^ ^177^ ^]^ DFT calculations support this mechanism by demonstrating strong binding energies between these materials and PF_6_^−^. However, conflicting reports suggest that strong binding to cations in the electrolyte also plays a crucial role. DFT calculations reveal that Li^+^ preferentially adsorbs onto lithiophilic sites, such as SrF_2_ (110) planes.^[^ ^178^ ^]^ Ab initio molecular dynamics simulations further indicate that Li^+^ migrates into defective regions in graphene oxide (GO), where these defects act as lithiophilic sites and exhibit a strong affinity for Li^+^.^[^ ^179^ ^]^ Phase‐field simulations confirm that these defect sites play a critical role in suppressing anisotropic Li growth. Experimentally, a combination of polar functional groups in polydopamine and GO, along with defects in the GO layers, enhances Na^+^ binding, improving electrolyte wettability and substrate adhesion. This, in turn, effectively mitigates Na dendrite growth (Figure 11F).^[^ ^180^ ^]^ Strong binding between the separator and anions in the electrolyte is often considered essential for achieving uniform deposition. However, successful cases have also been reported where strong binding to cations plays a significant role. Therefore, we encourage further combined computational and experimental studies to clarify this mechanism.

The separator also influences SEI formation and stability. By interacting with the SEI, it ensures uniform ion deposition, reduces dendrite growth, and extends cycle life.^[^ ^181^ ^]^ Separators with strong dipole moments attract electrons, suppressing solvent reduction and promoting the formation of a stable, inorganic SEI.^[^ ^182^ ^]^ To enhance this effect, carboxyl groups with high electronegativity are incorporated into the plant cellulose‐based separator framework, generating a strong dipole moment. This intensified dipole interaction facilitates the cleavage of P−F bonds in NaPF_6_, resulting in a NaF‐rich SEI. Moreover, by drawing in electrons, the separator prevents organic solvent reduction, thereby inhibiting the formation of organic oligomers in the SEI. Preloading separators with SEI‐forming species, such as F^−^ and NO_3_^−^, allows for a gradual and sustained release of these components, ensuring the continuous formation of a robust SEI with desirable characteristics, thereby enhancing long‐term protection.^[^ ^183^ ^]^

For safety, separators prevent direct anode‐cathode contact, reducing risks of short circuits and thermal runaway. Using flame‐retardant polymers, such as polyimide and polymerized 1,3‐dioxolane, as separators is a straightforward strategy to enhance overall safety.^[^ ^184^ ^]^ Flame‐retardant additives in the polymer matrix can help suppress combustion by reacting with electrolyte radicals.^[^ ^185^ ^]^ For instance, decabromodiphenyl ethane (DBDPE) additive acts as a gas‐phase flame retardant by generating HBr at high temperatures, while the incorporation of CaO additive promotes the formation of CaBr_2_, which provides liquid‐phase flame‐retardant properties.^[^ ^185^, ^186^ ^]^ Composite materials integrating polymers and inorganic enhance thermal resistance and stability. Incorporating thermally conductive materials, such as AlN and BN,^[^ ^186^, ^187^ ^]^ into separators facilitates heat management by absorbing and dissipating thermal energy, thereby preventing hotspots and improving thermal safety.^[^ ^186^ ^]^ The mechanical strength of the separator is as crucial as its flame retardancy.^[^ ^188^ ^]^ High mechanical strength is believed to prevent dendrite penetration.^[^ ^189^ ^]^ However, this claim is debated, as dendrites can still propagate through the pores of the separator. Nonetheless, enhancing mechanical strength remains beneficial. Adequate mechanical strength ensures that the separator can withstand the stresses caused by the significant volume expansion of alkali metal anodes, maintaining its structural integrity without breaking.^[^ ^190^ ^]^

There is a significant distinction in the application of separators for LMBs when compared to their Na and K counterparts. Polyolefin separators, including polypropylene (PP) and polyethylene (PE), are extensively utilized in LMBs, offering the advantages of cost‐effectiveness and ease of processing. Polyolefin is an affordable material that can be readily processed to the desired thickness and shape, and it is compatible with existing industrially scalable battery manufacturing processes. However, the direct use of polyolefin separators in SMBs and PMBs can result in rapid short‐circuiting. This discrepancy is due to the different mechanisms of dendrite growth. Li dendrites, which originate from the root of the nuclei, can be effectively impeded by the nanopores of polyolefin separators. In contrast, Na and K dendrites grow on the surface of the nuclei, often because of SEI breakdown. These dendrites form nanostructures that can propagate through the nanopores of polyolefin separators, leading to short circuits.^[^ ^191^ ^]^ To mitigate rapid short circuits, significantly thicker glass fiber separators are commonly employed in SMBs and PMBs. Nevertheless, separator functionalization that addresses dendrite‐related issues can render polyolefin separators viable for SMBs and PMBs. For example, polyolefin separators modified with polydopamine‐graphene heterostructures and MOFs have proven effectiveness for SMBs and PMBs, respectively.^[^ ^192^ ^]^

### 3.5 Solid‐State Electrolytes

Recent advances in the development of ionic conductor materials with conductivity comparable to that of organic liquid electrolytes make SSEs highly promising for enabling next‐generation metal anodes.^[^ ^193^ ^]^ SSEs are generally classified into inorganic solid‐state electrolytes and solid‐state polymer electrolytes.^[^ ^193^, ^194^ ^]^ The former exhibits high ionic conductivity at room temperature, a high shear modulus, and excellent mechanical strength.^[^ ^195^ ^]^ In contrast, solid polymer electrolytes excel in providing low interfacial resistance and good flexibility, making it less challenging to stabilize the interface between the SSE and electrode materials.^[^ ^196^ ^]^ There are two existing bottlenecks to enable high‐energy solid‐state batteries: the limited ionic conductivity of SSE, compared to liquid electrolytes, and the stabilization of the interface between the SSE and the anode.^[^ ^197^ ^]^ The latter challenge is exacerbated in alkali metal solid‐state batteries due to the low adhesion and high reactivity between the alkali metal and the SSE.^[^ ^194^, ^196^, ^198^ ^]^ Therefore, this section focuses on the existing challenges, as well as fundamental properties and strategies relevant to the design of Li/SSE and Na/SSE. Results relevant to the K/SSE interface are currently scarce, especially derived from computational studies.^[^ ^198^, ^199^ ^]^ Therefore, we discuss relevant strategies that can be transferable to this system from other alkali metals interfaces and recent advances in the development of K^+^ conductors.

**Figure 12:**
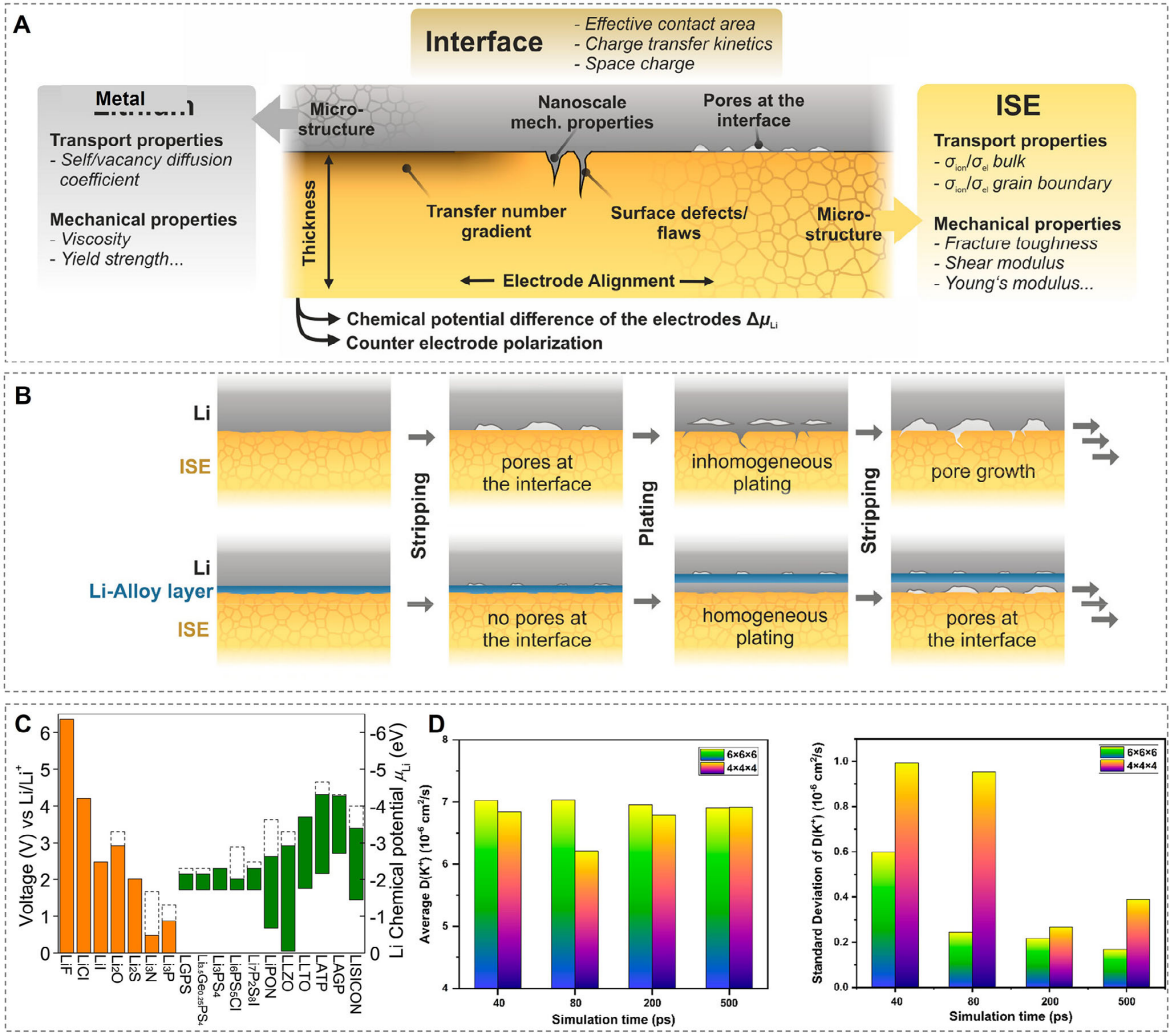
Key parameters in solid‐state electrolyte design. A) Overview of critical properties influencing Li penetration in fully dense SSEs, such as LLZO. B) Schematic representation of the morphological evolution during Li stripping and plating cycles in a Li|SSE half‐cell, with and without alloy interlayer modifications. (Reproduced with permission.^[^ ^201^ ^]^ Copyright 2020, American Chemical Society). C) Illustration of the electrochemical window of solid electrolytes, highlighting typical SEI components and their stability ranges. (Reproduced with permission.^[^ ^212^ ^]^ Copyright 2015, American Chemical Society). D) Average diffusion coefficients for K^+^ in Cl^−^doped K_3_SbS_4_ SSE, according to the molecular dynamics simulation length. (Reproduced with permission.^[^ ^218^ ^]^ Copyright 2024, American Chemical Society).

Short circuits caused by metal penetration, ultimately leading to dendritic growth, are still possible in alkali metal solid‐state batteries.^[^ ^199^, ^200^ ^]^ Krauskopf et al. summarized the key parameters that govern inhomogeneous Li growth through SSEs (**Figure** **12**A).^[^ ^201^ ^]^ From the perspective of the SSE/anode interface, defects, and microstructural features have a significant negative impact on short‐circuit susceptibility. The imperfect interface contact observed at the alkali metal/SSE interface, yields a heightened interface impedance and hinders the efficient transport of ions and electrochemical reactions involving alkali metal atoms at the interface.^[^ ^202^ ^]^ This issue is further exacerbated in inorganic solid‐state electrolytes due to the challenging interfacial compatibility and stability between the metal and the SSE.^[^ ^196^ ^]^ A comprehensive understanding of the alkali metal/SSE interface chemistry and its effect on the alkali metal deposition behavior is crucial to develop strategies for suppressing dendrite growth. Recent studies have highlighted the possibility of electron conduction through ceramic SSEs, leading to the formation of dendritic protrusions in grain boundary regions.^[^ ^203^ ^]^ Barai et al. employed a multiscale model combining DFT, AIMD, Monte Carlo simulations, and force‐field molecular dynamics to predict dendrite growth through LLZO.^[^ ^203a^ ^]^ The failure of LLZO at higher current densities was attributed to its inability to undergo plastic deformation, which is critical for delaying the initiation of Li dendrites in grain boundaries. Their findings show that the growth pattern of Li is closely related to the presence of pores and grain boundaries in the SSE. Recently, the grain boundary regions are pointed as a focal point for dislocation, directly preceding a dendrite in inorganic SSE, resulted from mechanical stress induced by dendrite expansion.^[^ ^204^ ^]^ The preferential dendritic growth along grain boundaries and voids on the SSE is no exclusive of Li solid‐state batteries, being also a problem in Na and K solid‐state batteries.^[^ ^199^, ^205^ ^]^

The critical current density on Na and K, which is the current in which dendrites form in a solid‐state cell is expected to be higher compared to Li cells due to the higher Na/K self‐diffusion on Na/K metal.^[^ ^62^ ^]^ However, the critical current density for the formation of voids can be lower, leading to a decrease in contact between the SSE and the metal anode and, subsequent dendrite formation.^[^ ^5^, ^83^ ^]^ The interface between sodium and Na_3.4_Zr_2_Si_2.4_P_0.6_O_12_ (NZP) was investigated in the work of Ortmann et al. revealing that current constrictions were caused by non‐ideal physical contact between Na and the SSE.^[^ ^206^ ^]^ This phenomenon was later confirmed in a zero‐excess Na configuration using the same SSE.^[^ ^207^ ^]^ One practical strategy to mitigate the morphological instabilities and sluggish diffusion kinetics of metal deposition at the metal/SSE interface, potentially avoiding the formation of dendrites, is the use of interlayers that promote chemical modifications at the interface. Fuchs et al. developed an analytical protocol to further investigate the microstructures of Li and Na at Cu/SSE, steel/SSE, and Al/SSE interfaces.^[^ ^208^ ^]^ By combining focused ion beam and electron backscatter diffraction techniques, the authors observed large grain sizes with grain boundaries predominantly oriented perpendicular to the interlayer/SSE interface, negatively impacting electrochemical performance. Pore formation was observed during discharge at the interface between the grain bulk and the SSE. Interestingly, no pore formation was detected at the intersection of grain boundaries and the interface, which was attributed to faster metal diffusion along grain boundaries.

Alloy‐forming interlayers, in particular, can enhance Li diffusivity towards the interface, reducing pore formation.^[^ ^68b^ ^]^ As a result, homogeneous plating can be achieved by preventing contact loss between the SSE and the electrode (Figure 12B). However, if metal diffusivity within the alloy layer is insufficient, metal atoms plate between the alloy layer and the SSE, leading to pore formation in subsequent cycles. Consequently, morphological stability is only improved during the initial cycles. Sandoval et al. investigated Li plating and stripping on 100‐nm silver and gold interfacial layers using electron microscopy, X‐ray microcomputed tomography, and mesoscale modeling.^[^ ^209^ ^]^ The alloy layers mitigated contact loss between the SSE and the interlayer metal, enabling uniform Li nucleation and growth, with alloy nanoparticles dispersed throughout the Li layer during deposition. In the study by Lowack et al., Zn, Ag, In, and Sn were tested as interlayers for zero‐excess Na cells with NASICON SSE. Among the strategies to improve physical contact between the current collector and the SSE, the use of seed layers, in particularly Sn, significantly reduced the electrochemical overpotential of Na deposition and improved the interface between the current collector and the SSE.^[^ ^210^ ^]^

The interphase formation can enhance the stability of the SSE, however, the decomposition products may exhibit undesirable conductivity, leading to the continuous degradation of the SSE. Computational simulations can be used to estimate properties relevant for defining the interfacial stability, such as electrochemical stability window and phase equilibrium, providing fundamental insights about the interphase formation mechanism. The electrochemical stability window of the Li superionic conductor Li₁₀GeP₂S₁₂ was estimated using a combination of DFT and AIMD simulations in the work of Mo et al.^[^ ^211^ ^]^ The results suggested that the high electrochemical stability window (>5 V) reported in earlier experimental studies is attributed to passivation reactions that form Li₂S and P₂S₂.^[^ ^193^, ^211^ ^]^ Further computational studies have explored the impact of interfacial decomposition on the electrochemical stability window of SSEs. Zhu et al. screened the electrochemical stability and decomposition reaction energies of various SSEs using first‐principles computational methods (Figure 12C).^[^ ^212^ ^]^ It was concluded that SSE decomposition is thermodynamically favorable at the applied potential, leading to the formation of interphases that extend the electrochemical stability window of the SSE. The stability of the SSE/Na interface was predicted across a range of SSEs by combining first‐principles methods and data‐mining approaches in the work of Lacivita et al.^[^ ^213^ ^]^ The Na conductors screened included aluminates, NASICONs, anti‐perovskites, sulfides/selenides, borohydrides, and halo‐aluminates. The authors suggested that the anodic stability of Na compounds can be enhanced compared to their lithium equivalent due to structural stabilization factors, such as the higher compatibility of Na^+^ with large anions. High voltage stability (>4 V) was predicted for phosphate‐based NaSICONs and the borohydride superionic conductor Na₂B₁₂H₁₂, owing to covalent stabilization. A comparison with Li‐containing materials revealed that Na compounds generally exhibit lower reduction and oxidation potentials than their Li counterparts.^[^ ^213^ ^]^ A comprehensive first‐principles assessment of the voltage windows and thermodynamic stability window, similar to the existing for Li/SSE and Na/SSE, is currently required for K ionic conductors to fully elucidate the specific interfacial stability of K/SSE systems. However, insights into the interfacial stability of K/SSE systems can be derived from studies on other alkali metal interfaces. For instance, β‐alumina solid electrolyte (BASE) has demonstrated improved cycling stability in K‐S cells and enhanced interfacial stability against Na metal. Therefore, a comparable performance of K‐BASE should be expected against K‐metal.^[^ ^198^, ^214^, ^215^ ^]^ The formation of Na₂S and Na₃P‐based interphases has been reported in the work of Wenzel et al. for the Na_3_PS_4_ conductor, which can be translated for K‐SSE containing sulfides, such as K₃SbS₄.^[^ ^198^, ^215^ ^]^

Recently, efforts have been made to overcome the limited availability of K‐SSEs with high ionic conductivity, which is currently in its early stages compared to Li and Na batteries.^[^ ^198^, ^216^ ^]^ The atomic layer deposition process for the K phosphorous oxynitride (KPON) films ion‐conductors was reported in the work of Nuwayhid et al. and compared with other alkali metals‐based (Li, Na) phosphorous oxynitride (APON).^[^ ^216b^ ^]^ The growth kinetics of KPON was similar to the NaPON, however, the strong reactivity of the precursor K‐*tert*‐butoxide led to a thin film with low N content, similar to K_3_PO_4_/K_2_CO_3_ film, and ionic conductivity higher than NaPON at room temperature. A new phase of K carbazolide (KC_12_H_8_N) was synthesized as a novel K‐SSE in the work of Guo et al., revealing high ionic conductivity and promising electrochemical stability window in comparison with other K^+^ conductors at the higher temperature of 373 K.^[^ ^217^ ^]^ The authors reported that the conductivity can be further optimized by adjusting the concentrations of tetrahydrofuran and K, also promoting enhanced interfacial stability with the K_2_S electrode during cycle. The current implementation of machine learning potentials plays a key role in increasing the scale of simulations, allowing researchers to overcome computational barriers and providing a viable route to guide the synthesis of new SSEs for K batteries. Zhang et al. investigated modifications to cubic K_3_SbS_4_ by conducting molecular dynamics simulations with neural network‐based potentials.^[^ ^218^ ^]^ Through simulations lasting 0.5 ns, the authors predicted that Cl doping induced the formation of K vacancies, thereby increasing the ionic conductivity of the SSE (Figure 12D).

## 4 Summary and Outlooks

Alkali metal batteries with the zero‐excess configuration demonstrate superior energy density compared to conventional ion batteries where there is an excessive amount of Li/Na/K. Despite significant progress in understanding fundamental mechanisms and electrochemical behaviors such as the anode reactions highlighted in this review, the practical application of ZEMBs still faces substantial challenges and requires further advancements. In general, anode‐excess and anode‐less batteries share a similar configuration, with the primary distinction being the amount of metal anode present. On the one hand, this similarity allows insights and findings from traditional metal batteries to serve as valuable references for studying anode‐less batteries, particularly regarding the formation and structural evolution of SEI and ion transport mechanisms. On the other hand, the reduced metal content in anode‐less batteries introduces significant differences that can make some of the conclusions less reliable or even irrelevant, especially regarding metrics like CE and cycle stability. In contrast, anode‐free batteries exhibit significant differences in configuration compared to anode‐excess batteries, as the active metal is replaced by a specially designed current collector serving as the “anode”. This design is expected to enhance the system's energy density further, but the absence of active metal on the anode side results in a lower tolerance for cation consumption caused by undesired reactions such as SEI fractures, dendrite formation, and dead metal accumulation. It significantly increases the complexity of anode design and emphasizes the need for optimization strategies to achieve high CE.

**Figure 13:**
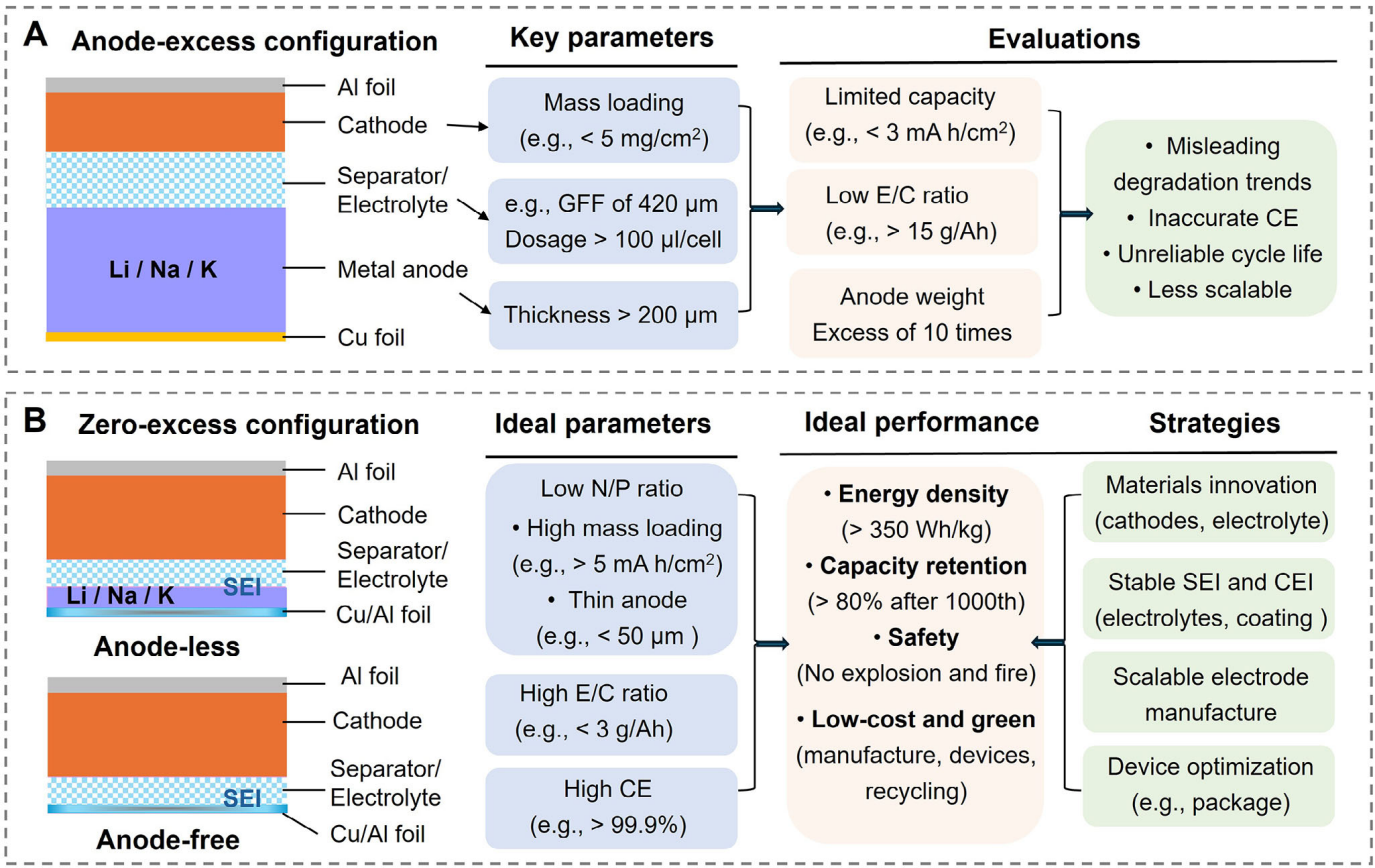
Comparison of anode‐excess and zero‐excess configurations. A) Configuration, key parameters, and performance evaluations of typical anode‐excess cells, emphasizing their high metal anode thickness, excess electrolyte usage, and cycling performance. B) Configuration, key parameters, target performances, and optimization strategies for zero‐excess configuration cells, highlighting their design considerations for energy density, cycling stability, and manufacturing scalability.

A comparison between typical anode‐excess and zero‐excess configurations is summarized in **Figure** **13**. In a coin cell setup, a metal battery typically consists of a low‐mass‐loading cathode (e.g., <5 mg cm^−^^2^), a thick separator (e.g., >200 µm), a thick metal anode (e.g., >200 µm) and an excess amount of electrolyte. This setup results in a limited areal capacity (e.g., < 3 mAh cm^−^^2^), a low electrolyte‐to‐cathode (E/C) ratio, and excessive use of metal (Figure 13A). While cells shown in the literatures exhibit impressive cycle life of over 1000 or even 10 000 cycles, the capacity degradation trends observed in these cells differ significantly from those under real‐world conditions, prompting the issue of the lack of practical references. Furthermore, the scalability and stability of optimization strategies for alkali metal anodes are often proposed based on the anode‐excess cell configuration and thus require rigorous evaluation to ensure viability in realistic scenarios.

For the practical application of ZEMBs, performance requirements can be stringent. Energy density, cycling life, safety, and cost‐effectiveness demand the most attention and innovative solutions. Ideally, the energy density target is ≈350 Wh kg^−1^ and 750 Wh L^−1^, and cycling life should exceed 1000 cycles with a capacity retention of over 80%.^[^ ^219^ ^]^ Achieving these targets necessitates the optimization of key parameters such as N/P ratio, E/C ratio, and CE (Figure 13B). For anode‐less configurations, a low N/P ratio in metal batteries is essential for achieving high energy density but also poses significant performance challenges, such as reduced cycling life. Using ultrathin alkali metal foils (e.g., 20 µm) can enhance energy density; however, manufacturing and handling high‐quality, ultrathin metal foils remain challenging. As a result, the recommended N/P ratio in LMBs is ≈2.5, which corresponds to a 50 µm thick foil for an areal capacity of 4 mAh cm⁻^2^, achieving an energy density above 300 Wh kg^−1^.^[^ ^220^ ^]^ Given that Na and K have lower capacities and are more chemically reactive and mechanically softer than Li, the thickness of Na and K should be greater than that of Li. The application of SMBs and PMBs can be explored progressively, starting with a thickness below 100 µm. For the anode‐free configuration, the cathode mass loading is expected to be 20–30 mg cm⁻^2^, providing an areal capacity of 4–6 mAh cm⁻^2^.^[^ ^221^ ^]^ Simultaneously, the E/C ratio should approach the level of commercial LIBs, ≈1.0–1.5 g Ah⁻¹.^[^ ^221^ ^]^ While some relaxation may be necessary to account for electrolyte consumption during SEI formation, the E/C ratio should not exceed 3 g Ah⁻¹.^[^ ^221^, ^222^ ^]^ Second, a high CE, particularly initial CE, is crucial for achieving cycle stability. Assuming a zero‐excess metal configuration, a CE of 99.98% is required to maintain a capacity retention of over 80% after 1000 cycles. This is calculated using the formula:

(2) $$CE=e\frac{\ln\left(R\right)}{n}$$

where *R* is the retention ratio and *n* is the cycle number. In the case of excessive metal, the CE formula is adjusted to account for the additional capacity provided by the excess. The revised capacity retention R′ is calculated as:

(3) $$R^{\prime }=1-\frac{1}{f}\left(1-R\right)$$

where *f* is the excess factor (e.g., f = 3 for 200% excess metal). Finally, safety and cost‐effectiveness are pivotal for the practical deployment of ZEMBs. Considering the high reactivity of alkali metals, minimizing safety risks is paramount, as extensively discussed in several reviews.^[^ ^6^, ^223^ ^]^ Cost‐effectiveness must also be ensured through scalable and efficient manufacturing processes for all cell components.

Accordingly, to achieve the set parameters and performance, we highlight a few promising strategies including materials innovation, interfacial reactions, scalable manufacturing, and device optimizations. While general approaches such as electrolyte design, artificial SEIs, and 3D scaffolds are applicable to all Li, Na, and K metal batteries, their specific implementations must be carefully tailored to the unique physicochemical properties of each metal to realize zero‐excess conditions.

1. Materials innovation: Developing novel materials is critical to achieving high energy density and stable cycling performance. This should include discovering advanced cathode materials and exploring emerging systems such as metal‐S batteries and solid‐state batteries. Advances in simulations such as generative modeling or inverse design are promising avenues to aid future experiments, identifying the best candidates in silico that can then be experimentally verified and compared to existing solutions. The last step is currently crucial, as model hallucinations and the difficulty of identifying the true novelty of generated structures need to be overcome. An example of this is the application of deep learning to predict the viscosity of ionic liquids at room temperature.^[^ ^224^ ^]^ By utilizing a cleaned dataset, the authors not only achieved accurate viscosity predictions but also identified key molecular descriptors associated with high‐performing candidates. These insights can guide experimental studies that focus on the identified descriptors to validate model predictions. The need for experimental validation extends to other areas predominantly explored through computational studies, such as the calculation of diffusion parameters for various transport mechanisms. Strengthening collaboration between theory and experiment would be particularly valuable for artificial SEIs, where defect engineering can be strategically applied, and optimal transport behavior can be predicted through simulations of single‐component systems.
2. Interfacial reactions: SEI, in the case of cathode, i.e., cathode‐electrolyte interphase (CEI), is the key factor in determining performance. Strategies must address undesired reactions such as dendrite formation, SEI fractures, and side reactions, which can compromise efficiency and safety. From the experimental perspective, techniques suitable for in situ investigation of properties are emerging and will generate in‐depth and representative insights into the cycling mechanisms at a battery cell's operational condition. From the computational perspective, increasing computing power can enhance the capability of simulating a high number of parameters of the models. Especially the use of universal machine learning force fields has the potential to remove the need for resource‐intensive training data generation for MD calculations. New approaches need to be thoroughly tested, and results need to be counterchecked by experiments to ensure the applicability for specific domains such as ZEMBs.
3. Computational‐driven design: The current state‐of‐the‐art for modeling work related to energy materials, in terms of accuracy, is the application of plane‐wave DFT and AIMD. However, these methodologies generally lack the capability to scale computational models into sizes larger than a few nanometers and simulate length times longer than the picoseconds scale. Larger scale simulations are needed to obtain fundamental properties and observe the dynamics at the electrolyte/anode interface, in which modeling works are close to nonexistent for alkali metals other than Li. While properties of liquid electrolytes can often be obtained through classical MD, there are no established classical force fields that can be universally applied to metals and solid‐state electrolytes. Recent advancements in the development of machine learning interatomic potentials (MLIPs) are overcoming this limitation, enabling simulations using computational models spanning several nanometers and molecular dynamics simulations reaching the nanosecond scale, while retaining near DFT accuracy. There are three primary approaches to implementing MLIPs in simulations: generating the potential during the simulation by coupling on‐the‐fly machine learning with MD; training an MLIP from scratch using a specific dataset; or employing an existing universal MLIP. These computational techniques have already been used to support the design of novel solid‐state electrolytes, which have previously been unfeasible using purely DFT and AIMD. However, obtaining fundamental insights into phenomena such as SEI formation reactivity and alkali metal nucleation dynamics at the interface remains challenging for these methodologies. This is primarily due to the numerous interactions occurring at the electrolyte/anode interface, which still require significant computational effort for on‐the‐fly MLIPs and a substantial dataset to train an MLIP from scratch. Universal MLIPs offer an out‐of‐the‐box solution for modelling these reactions; however, since they are typically trained on datasets containing stable equilibrium materials structures across the periodic table, their performance in high‐reactivity scenarios, i.e., involving non‐equilibrium transformations, has yet to be thoroughly evaluated.
4. Scalable manufacturing of electrodes: For cathodes, increasing mass loading can enhance energy density but also introduce challenges, such as maintaining structural consistency in thick electrodes, uneven mixing of components, fractures, and delamination during slurry preparation, drying, and calendaring processes. Additionally, mass transport in thick electrodes is more complex, requiring optimization of porosity, tortuosity, wettability, and ion diffusion. For anodes, manufacturing high‐quality metal anodes remains challenging. Innovations should address the problems of precise thickness control, reducing surface roughness, resistance to oxidation in an ambient environment, and continuous processing to avoid safety risks during storage and transport.^[^ ^97b^ ^]^ Developing robust artificial SEI layers during anode progression is also essential.

Anode‐free configuration eliminates the need of alkali metal, which can simplify the manufacturing process and enable production under less restrictive environmental conditions. However, it requires scalable and low‐cost substrate production. Currently, Cu foil is the preferred substrate for alkali metal deposition due to its excellent mechanical properties (e.g., tensile strength, ductility), mature processing methods, and stable chemical and electrochemical properties. Note that Na and K will not form an alloy with Al, offering the possibility to use cheap Al to replace expensive Cu in Na and K metal batteries. Challenges in producing high‐quality Cu/Al foil include achieving low thickness (e.g., 6 µm), optimizing surface properties (e.g., specific crystal orientation, low surface roughness, and suitable modification layers), and addressing defect‐related issues.^[^ ^225^ ^]^

Efforts should focus on examining and mitigating the influence of defects on metal plating/stripping, implementing effective defect detection methods during pressing and maintaining surface quality throughout the anode production stages. Understanding the interplay between materials innovation, interfacial optimization, and scalable manufacturing techniques is of paramount importance for advancing alkali metal batteries.

5) Device optimization: It is a crucial aspect, as it directly impacts the energy density and safety of ZEMBs. Enhancing energy density involves improving the ratio of active components to inactive components within the cell device. For example, increasing the electrode‐to‐current collector ratio by optimizing material usage can significantly reduce the weight and volume of inactive materials, thereby boosting overall energy density. Utilizing low‐mass separators and innovative structural designs, such as blade batteries, can further contribute to improving the active‐to‐inactive component ratio. These approaches not only enhance the specific energy of the battery but also point to creating lightweight and compact energy storage systems.

Ensuring the safety and reliability of the device is another crucial aspect. It can be achieved through the integration of fire‐resistant packaging materials that reduce the risk of thermal runaway and improve the battery's resilience under extreme conditions. Advanced heat‐management systems are essential for maintaining a stable operating temperature, particularly in high‐power applications where heat generation is significant. Incorporating a battery auto‐close protection system can further enhance safety by automatically isolating the battery in the event of overheating, short circuiting, or mechanical damage. Combining strategies to improve energy density with robust safety mechanisms, device optimization paves the way for the practical deployment of high‐performance, safe, and reliable ZEMBs across a range of applications.

## Conflict of Interest

The authors declare no conflict of interest.
